# Discovery of novel furanylbenzamide inhibitors that target oncogenic tyrosine phosphatase SHP2 in leukemia cells

**DOI:** 10.1016/j.jbc.2021.101477

**Published:** 2021-12-10

**Authors:** Dhanya Raveendra-Panickar, Darren Finlay, Fabiana Izidro Layng, Lester J. Lambert, Maria Celeridad, Ming Zhao, Karina Barbosa, Laurent J.S. De Backer, Elizabeth Kwong, Palak Gosalia, Socorro Rodiles, John Holleran, Robert Ardecky, Stefan Grotegut, Steven Olson, John H. Hutchinson, Elena B. Pasquale, Kristiina Vuori, Aniruddha J. Deshpande, Nicholas D.P. Cosford, Lutz Tautz

**Affiliations:** 1 NCI-Designated Cancer Center, Sanford Burnham Prebys Medical Discovery Institute, La Jolla, California, USA; 2 Conrad Prebys Center for Chemical Genomics, Sanford Burnham Prebys Medical Discovery Institute, La Jolla, California, USA

**Keywords:** AML, breast cancer, chemical biology, leukemia, protein tyrosine phosphatase, PTPN11, SHP2, small molecule, inhibitor, anticancer drug, AIC, Akaike's Information Criterion, AICc, second-order corrected Akaike’s Information Criterion, AML, acute myeloid leukemia, DIEA, *N*,*N*-diisopropylethylamine, DiFMUP, 6,8-difluoro-4-methylumbelliferyl phosphate, DMF, *N*,*N*-dimethylformamide, DMSO, dimethyl sulfoxide, EDC, 1-ethyl-3-(3-dimethylaminopropyl)carbodiimide, ERK, extracellular signal–regulated kinase, ESI, electrospray ionization, EtOAc, ethyl acetate, FBS, fetal bovine serum, FGFR, fibroblast growth factor receptor, HOBt, hydroxybenzotriazole, IRS-1, insulin receptor substrate 1, N-SH2, N-terminal SH2 domain, OMFP, 3-O-methylfluorescein phosphate, p-ERK1/2, phospho-ERK1/2, PTP, protein tyrosine phosphatase, PTS, protein thermal shift, pTyr, phosphotyrosine, RFU, relative fluorescence unit, RT, room temperature, RTK, receptor tyrosine kinase, SAR, structure–activity relationship, sgRNA, single-guide RNA, SH2, Src-homology 2, SHP2, Src-homology 2 domain–containing phosphatase 2, SHP2cat, SHP2 catalytic domain, SHP2-WT, full-length SHP2 WT, STEP, striatal-enriched tyrosine phosphatase, TBS-T, Tris-buffered saline–Tween-20, THF, tetrahydrofuran, TNBC, triple-negative breast cancer

## Abstract

Disturbance of the dynamic balance between tyrosine phosphorylation and dephosphorylation of signaling molecules, controlled by protein tyrosine kinases and protein tyrosine phosphatases (PTPs), is known to lead to the development of cancer. While most approved targeted cancer therapies are tyrosine kinase inhibitors, PTPs have long been stigmatized as undruggable and have only recently gained renewed attention in drug discovery. One PTP target is the Src-homology 2 domain–containing phosphatase 2 (SHP2). SHP2 is implicated in tumor initiation, progression, metastasis, and treatment resistance, primarily because of its role as a signaling nexus of the extracellular signal–regulated kinase pathway, acting upstream of the small GTPase Ras. Efforts to develop small molecules that target SHP2 are ongoing, and several SHP2 allosteric inhibitors are currently in clinical trials for the treatment of solid tumors. However, while the reported allosteric inhibitors are highly effective against cells expressing WT SHP2, none have significant activity against the most frequent oncogenic SHP2 variants that drive leukemogenesis in several juvenile and acute leukemias. Here, we report the discovery of novel furanylbenzamide molecules as inhibitors of both WT and oncogenic SHP2. Importantly, these inhibitors readily cross cell membranes, bind and inhibit SHP2 under physiological conditions, and effectively decrease the growth of cancer cells, including triple-negative breast cancer cells, acute myeloid leukemia cells expressing either WT or oncogenic SHP2, and patient-derived acute myeloid leukemia cells. These novel compounds are effective chemical probes of active SHP2 and may serve as starting points for therapeutics targeting WT or mutant SHP2 in cancer.

Protein tyrosine phosphorylation is a reversible post-translational modification that controls and fine-tunes cellular responses to a wide variety of extracellular and intracellular stimuli (1). Dysregulation of the delicate balance between phosphorylation and dephosphorylation of signaling molecules, mediated by protein tyrosine kinases and protein tyrosine phosphatases (PTPs), respectively, is a distinctive feature of many cancers (2). Thus, it is not surprising that the discovery of agents to restore this balance has been the focus of many anticancer efforts. However, it is remarkable that while most Food and Drug Administration–approved targeted cancer drugs are tyrosine kinase inhibitors, currently there are no Food and Drug Administration–approved PTP inhibitors (3, 4). Recent evidence suggests that members of the PTP enzyme family are also promising drug targets for cancer therapy (5, 6, 7, 8). A prime target is the Src-homology 2 (SH2) domain–containing phosphatase 2 (SHP2, *PTPN11*), a crucial positive regulator of receptor tyrosine kinase (RTK)–driven signaling in response to growth factors and cytokines, including signaling through the Ras/RAF/extracellular signal–regulated kinase (ERK), the PI3K/Akt, and the JAK/STAT pathways (9, 10, 11, 12). SHP2 activity is tightly regulated in normal cells (Fig. 1, *A* and *D*) (13). Under resting conditions, SHP2 adopts a “closed” autoinhibited conformation, in which the N-terminal SH2 domain (N-SH2) blocks access to the active site in the phosphatase domain. Upon RTK activation, SHP2 is recruited by tyrosine phosphorylated motifs within either RTK cytoplasmatic regions or adapter and scaffolding proteins *via* its two SH2 domains, resulting in a conformational switch that activates SHP2 by rendering the active site accessible to its substrates (“open” active conformation). Hyperactive SHP2 is associated with tumorigenesis, tumor maintenance, metastasis, and survival, as well as intrinsic and acquired resistance to targeted cancer drugs (14, 15, 16). Germline gain-of-function mutations in SHP2 that destabilize its autoinhibited conformation were first observed in ∼50% of cases of Noonan syndrome, a developmental disorder with increased risk of malignancy (17). Numerous somatic gain-of-function mutations that similarly cause a constitutive activation of SHP2 are primarily found in leukemias (18, 19, 20). In solid tumors, SHP2 activity is often enhanced *via* amplification or overexpression of growth factors, RTKs, or scaffolding adapters (16). Interestingly, cancers driven by certain Ras mutations such as KRas-G12C also depend on SHP2 activity, which promotes GDP/GTP cycling (21). Finally, SHP2 is also important for immune checkpoint function through modulation of programmed cell death 1, cytotoxic T lymphocyte–associated antigen 1, and B and T lymphocyte attenuator signaling, suggesting a potential immunotherapy based on targeting SHP2 (22). Together, these data demonstrate a clear link between SHP2 signaling and cancer, confirming that SHP2 is a key target for drug discovery and development.

**Figure 1:**
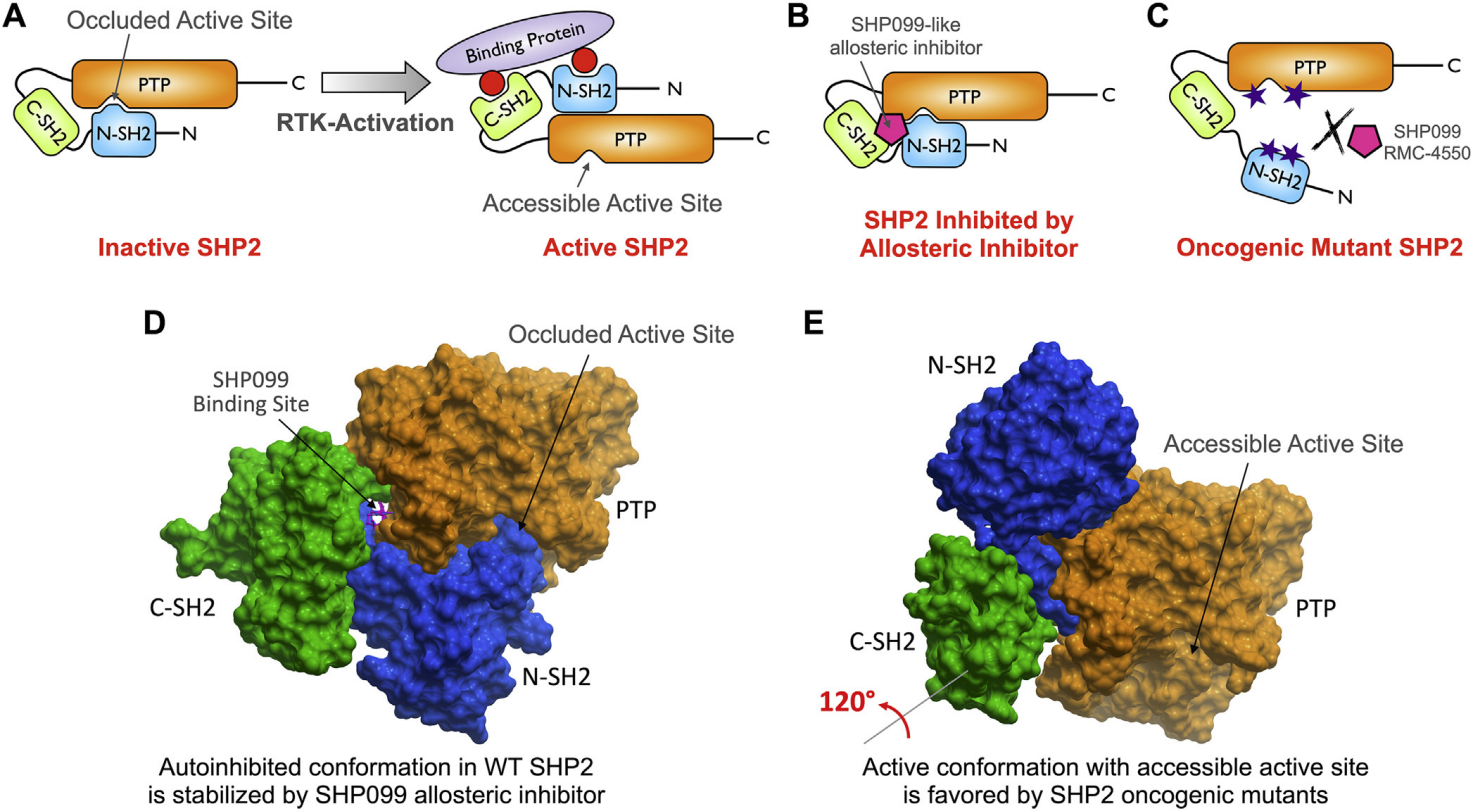
**SHP2 regulation, inhibition, and oncogenic mutations.***A*, model of SHP2 activation. Receptor tyrosine kinase (RTK) activation leads to SHP2 recruitment by tyrosine phosphorylated motifs *via* its two SH2 domains, resulting in a conformational switch from the “closed” autoinhibited to the “open” active conformation. *B*, model of SHP2 inhibition by allosteric inhibitor, such as SHP099. These “molecular glue”–type compounds stabilize the SHP2 inactive closed conformation. *C*, model of the common mechanism of SHP2 oncogenic variant activation. A single amino-acid mutation at the N-SH2/PTP domain interface prevents the intramolecular binding of the two domains, resulting in a constitutively active SHP2. Allosteric inhibitors such as SHP099 and RMC-4550 are not able to bind the open conformation preferred by the SHP2 gain-of-function mutants. *D*, crystal structure of WT SHP2 representing the closed and autoinhibited conformation as described in (*A*) (Protein Data Bank ID: 5EHR). N-SH2 domain, *blue*; C-SH2 domain, *green*; PTP domain, *orange*; the allosteric inhibitor SHP099 (*magenta*, *stick representation*) binds to a channel formed by the PTP, N-SH2, and C-SH2 domains and stabilizes the inactive conformation as described in *B*. *E*, crystal structure of the SHP2 E76K gain-of-function mutant in the open and active conformation (Protein Data Bank ID: 6CRF). To adopt this conformation, the C-SH2 domain rotates by ∼120° and thereby translocates the N-SH2 domain away from the active site. SHP099-like allosteric inhibitors cannot effectively bind this active conformation as described in *C*. C-SH2, C-terminal SH2; N-SH2, N-terminal SH2 domain; PTP, protein tyrosine phosphatase; SH2, Src-homology 2; SHP2, Src-homology 2 domain–containing phosphatase 2.

Targeting tyrosine phosphatases with small-molecule inhibitors has been a challenge historically, because the active site of PTPs is both highly conserved and highly charged. Inhibitors that bind to the active site are often potent but exhibit poor selectivity and limited cell membrane permeability (23, 24, 25, 26). This has led to the stigmatization of these enzymes as undruggable (27). Early efforts to therapeutically target SHP2 focused on inhibitors that bind in the active site (28). Most of these efforts failed to yield compounds that combine potency and selectivity for SHP2 with efficacy in cellular models (29). The first truly selective SHP2 small-molecule inhibitor with good cellular and *in vivo* efficacy, SHP099, was reported by Novartis in 2016 (30). Since then, a number of SHP099-like inhibitors have been reported, and several compounds in this class are currently in clinical trials for the treatment of solid tumors with elevated RTK signaling and certain K-Ras mutations. All these compounds share a common allosteric mechanism by which they stabilize the autoinhibited conformation of SHP2 and thereby prevent recruitment and activation of the phosphatase (31, 32). Specifically, the SHP099-like inhibitors act as a “molecular glue” by binding to a channel that is formed by the SHP2 phosphatase (or PTP) domain and its two SH2 domains, thereby locking SHP2 in the inactive conformation (Fig. 1*B*) (33). Because this binding channel is only present in the inactive state, the SHP099-like compounds exhibit potencies that are reduced by several orders of magnitude toward many of the frequently occurring gain-of-function mutants, in which a single point mutation disturbs the SHP2 autoinhibited conformation, resulting in constitutive activation (Fig. 1, *C* and *E*). Moreover, a recent report from Novartis demonstrated that fibroblast growth factor receptor (FGFR)–driven cancers, such as many breast or prostate cancers, are inherently resistant to SHP099-like inhibitors (34), because rapid feedback activation of the FGFR causes increased recruitment and activation of SHP2. Clearly, there is a need for next-generation SHP2 inhibitors that are effective against cancers driven by either SHP2 oncogenic mutants or aberrant signaling through the FGFR. Here, we describe the discovery of a series of furanylbenzamide-based inhibitors of both WT and oncogenic mutant SHP2. These compounds readily cross cell membranes and bind and inhibit SHP2 under physiological conditions. Our best inhibitors reduce the growth of various cancer cells, including patient-derived leukemia cells, at low micromolar concentrations.

## Results

### Discovery of furanylbenzamides as SHP2 inhibitors and synthesis of analogs

To identify novel SHP2 inhibitor scaffolds, we screened an in-house small-molecule library collection against one of the most frequent SHP2 oncogenic variants, E76K, using a protein thermal shift (PTS) assay (to be published elsewhere). Confirmed PTS hits were then subjected to *in vitro* phosphatase inhibition assays using SHP2-E76K. Among the identified hits was SBI-4668 (Table 1, #02), which showed good inhibitory activity against SHP2-E76K with an IC_50_ value of 1.8 μM. This compound was selected for structure–activity relationship (SAR) and mechanistic studies. Interestingly, the benzothiophenone-furanylbenzamide scaffold of SBI-4668 lacked an obvious phosphotyrosine (pTyr)-mimicking group and did not contain any other charged moiety that may reduce cell membrane permeability. Importantly, compounds in this series did not act as covalent inhibitors *via* Michael addition to the enone double bond (as discussed in more detail later).

**Table 1:** SAR of furanylbenzamide inhibitors and analogs

Compound number	Substance ID	Structure	SHP2-E76K; IC_50_, μM	SHP2-WT; IC_50_, μM	SHP2cat; IC_50_, μM
01	SBI-2130		0.48	5.0	0.22
02	SBI-4668		1.8	15	0.73
03	SBI-3192		2.3	4.1	1.1
04	SBI-3405		4.1	4.2	2.0
05	SBI-3404		4.2	11	1.4
06	SBI-3191		6.0	24	7.4
07	SBI-0165		7.2	13	2.4
08	SBI-2126		7.2	18	5.0
09	SBI-3204		10	15	4.5
10	SBI-6999		11	13	10
11	SBI-2128		11	14	3.1
12	SBI-2131		15	52	4.3
13	SBI-0287		20	53	ND
14	SBI-3194		39	56	ND
15	SBI-5923		52	62	ND
16	SBI-2129		65	>100	ND
17	SBI-4167		79	>100	ND
18	SBI-2349		84	87	ND
19	SBI-2124		91	>100	ND
20	SBI-9639		>100	>100	ND
21	SBI-4232		>100	>100	ND
22	SBI-1457		>100	>100	ND
23	SBI-2125		>100	>100	ND
24	SBI-2348		>100	>100	ND
25	SBI-3570		>100	>100	ND

Abbreviation: ND, not determined.

**Figure 2:**
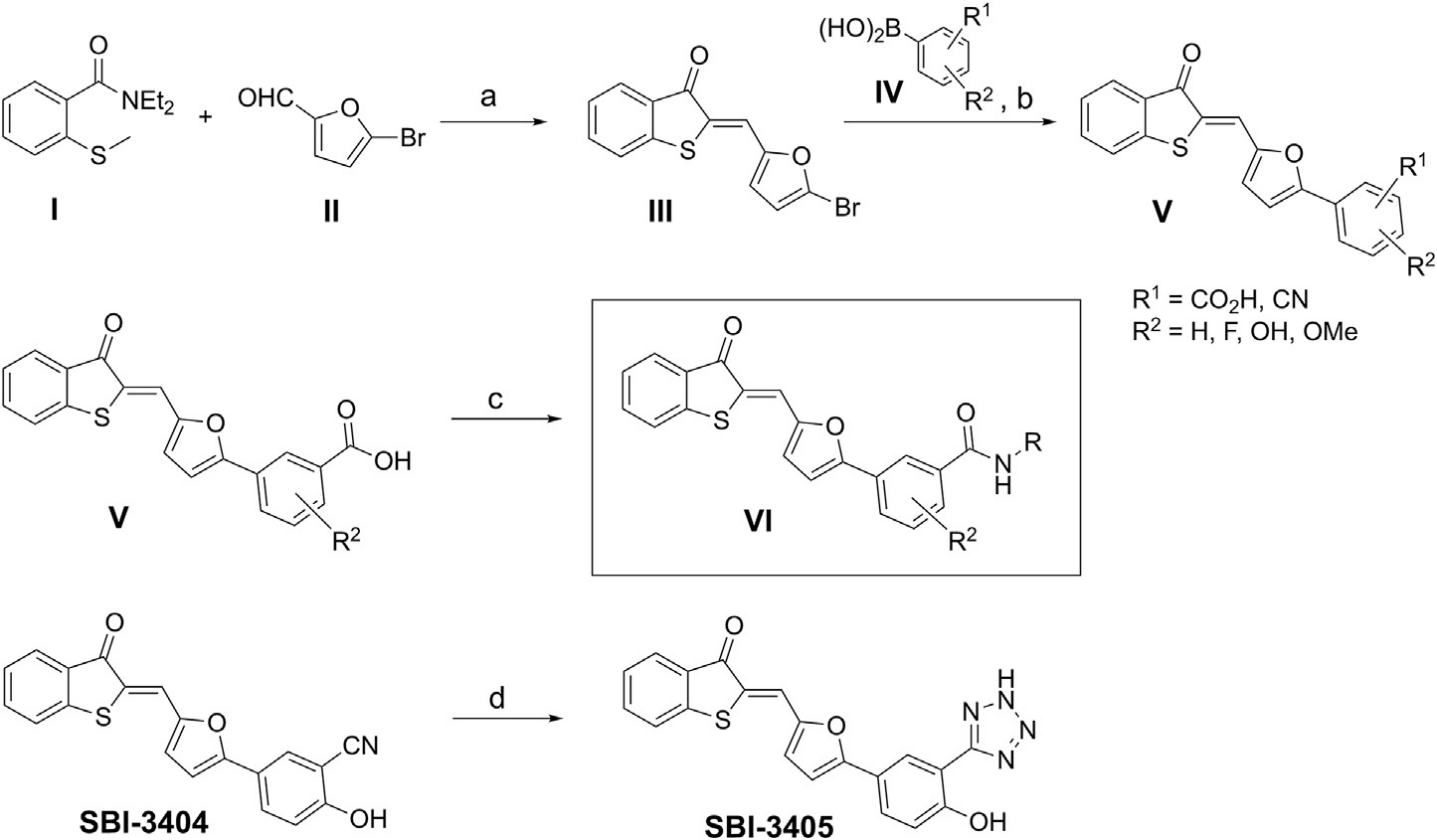
**General synthetic scheme to access benzothiophenone-furanylbenzamides and analogs.***α*-Lithiation of *N,N*-diethyl-2-(methylthio)benzamide I with LDA in dry THF followed by intramolecular alkylation and displacement of the diethylamide group afforded the benzothiophen-3-one *in situ*, which underwent an aldol condensation with the aldehyde II to deliver intermediate III. III was then subjected to Suzuki cross-coupling reaction with appropriate arylboronic acids IV to provide the target analogs V. The desired benzamides 6 were prepared by treating the benzoic acid derivatives V with appropriate amines in the presence of EDCI and HOBt in *N,N*-dimethylformamide. To afford the derivative in which the amide group is replaced with a tetrazole, benzonitrile SBI-3404 was heated with sodium azide in the presence of ammonium chloride in *N,N*-dimethylformamide to afford analog SBI-3405. a, LDA, THF, room temperature 0 °C, and 2 h. b, Pd(PPh_3_)_4_, 2 M Na_2_CO_3_, DME, 80 °C, 6 to 12 h. c, HOBt, EDC.HCl, R-NH_2_, Et_3_N, DMF, room temperature, 3 h. d, NaN_3_, NH_4_Cl, DMF, 100 °C, and 1 h. DME, dimethoxyethane; DMF, *N*,*N*-dimethylformamide; EDCI, 1-ethyl-3-(3-dimethylaminopropyl)carbodiimide; HOBt, hydroxybenzotriazole; LDA, lithium diisopropylamide; THF, tetrahydrofuran.

Using the general synthetic strategy shown in Figure 2, we synthesized the original hit, SBI-4668, and a total of 24 related analogs for subsequent testing in SHP2 phosphatase assays (Table 1). The first step to access key intermediate III involved reacting equimolar quantities of *N,N*-diethyl-2-(methylthio)benzamide I and 5-bromo-2-furaldehyde II using a previously described procedure with minor modifications (35). The resulting intermediate III was subjected to Suzuki cross-coupling reaction conditions with the appropriate arylboronic acids IV to provide the target analogs V. The desired benzamides VI were prepared from the corresponding benzoic acid derivatives V using standard peptide coupling with appropriate amines. The tetrazole derivative SBI-3405 was accessible *via* azide cycloaddition from benzonitrile SBI-3404. All compounds had a purity of >95%.

### SAR and biochemical evaluation of furanylbenzamide SHP inhibitors

To test the synthesized compounds for their potential to inhibit SHP2 activity, a fluorescence intensity-based assay using 6,8-difluoro-4-methylumbelliferyl phosphate (DiFMUP) as the substrate was adapted for three recombinant SHP2 constructs: (1) the SHP2 catalytic domain (SHP2cat; residues 248–527), (2) the full-length SHP2-E76K oncogenic mutant, and (3) the full-length SHP2 WT (SHP2-WT). The recombinant SHP2 proteins were expressed and purified as described previously (36). A dually phosphorylated peptide derived from the insulin receptor substrate 1 (IRS-1) served as a surrogate binding protein and was used to activate SHP2-WT (30, 36). The constitutively active E76K mutant did not require activation. Similarly, the SHP2cat construct, which lacks the SH2 domains, did not need to be activated. Michaelis–Menten experiments to determine the DiFMUP Michaelis–Menten constant (*K*_*m*_) for each SHP2 construct were performed as described previously (36) and yielded the following values: SHP2-WT, *K*_*m*_ = 60 μM; SHP2-E76K, *K*_*m*_ = 20 μM; and SHP2cat, *K*_*m*_ = 20 μM. Relative maximum rates (*V*_max_) of the DiFMUP reactions expressed as relative fluorescence units per minute (RFU/min) were as follows: SHP2-WT, *V*_max_ = 871 RFU/min; SHP2-E76K, *V*_max_ = 2730 RFU/min; and SHP2cat, *V*_max_ = 2912 RFU/min. IC_50_ values for each compound were determined from initial rates in 10-point dose–response assays using DiFMUP at a concentration corresponding to its *K*_*m*_ value for the respective SHP2 construct. The most active compounds exhibited submicromolar or low micromolar activity against SHP2 (Table 1). Active compounds generally inhibited all three SHP2 constructs, suggesting that they act on the SHP2cat. Among the various furanylbenzamide analogs we generated, compounds containing an *N*-phenyl-2-hydroxybenzamide moiety showed the greatest potency, including the most potent inhibitor SBI-2130 (#01, E76K IC_50_ = 0.48 μM), the original hit SBI-4668 (#02, E76K IC_50_ = 1.8 μM), and SBI-3192 (#03, E76K IC_50_ = 2.3 μM). Interestingly, deletion of the benzothiophenone group completely abrogated inhibitory activity (#21, E76K IC_50_ > 100 μM), suggesting a crucial role of the benzothiophenone for binding to SHP2. However, the benzothiophenone moiety could be replaced with a cyclopentanone without any loss of activity (#03, E76K IC_50_ = 2.3 μM). This indicates that the presence of the 5-membered ring and carbonyl oxygen is sufficient to retain activity. The carboxylic acid derivative (#10, E76K IC_50_ = 11 μM) was sixfold less potent than the phenyl amide #02, though still tolerated. Replacements for the benzothiophenone were explored using analog #10. Thus, the indanone (#09; E76K IC_50_ = 10 μM) was similar in potency, whereas both the benzofuranone (#07; E76K IC_50_ = 7.2 μM) and cyclopentanone (#06; E76K IC_50_ = 6.0 μM) were slightly more potent. Interestingly, the cyclohexanone analog (#14; E76K IC_50_ = 39 μM) was sixfold less active than the corresponding cyclopentanone derivative #06.

We next focused our SAR studies on the benzamide ring. Deletion of the *N*-phenyl amide group (#17, E76K IC_50_ = 79 μM) resulted in a ∼165-fold loss in potency compared with the most potent compound #01. However, replacement of the amide group with a cyano group (#05, E76K IC_50_ = 4.2 μM) or tetrazole moiety (#04, E76K IC_50_ = 4.1 μM) was better tolerated and only resulted in a modest decrease in potency. Deletion of both the amide and hydroxyl groups completely abolished activity (#25, E76K IC_50_ > 100 μM), suggesting a role of the hydroxyl group for inhibition. Indeed, we found that the hydroxyl group is essential for activity, as its deletion (#15, E76K IC_50_ = 52 μM) or replacement with either methoxy (#18, E76K IC_50_ = 84 μM) or fluoro (#24, E76K IC_50_ > 100 μM) greatly diminished the inhibitory activity of analogs. Several different *N*-substituted amides were synthesized and tested. We found that potency was substantially reduced when the *N*-phenyl moiety was replaced by *N*-benzyl (#16, E76K IC_50_ = 65 μM). On the other hand, inhibition of SHP2 was increased, compared with the original hit, when the *N*-phenyl was substituted with *N*-4-aminophenyl (SBI-2130, E76K IC_50_ = 0.48 μM). Moving the amino group into the 3-position reduced the activity (#08, E76K IC_50_ = 7.2 μM). Other *N*-substituents tested were either not tolerated, including piperazine (#19, E76K IC_50_ = 91 μM), 4-methylpiperazine (#23, E76K IC_50_ > 100 μM), and 2-aminoethanol (#22, E76K IC_50_ > 100 μM), or exhibited substantially lower potency, including phenylhydrazine (#11, E76K IC_50_ = 11 μM), piperidine (#12, E76K IC_50_ = 15 μM), and isopropyl (#13, E76K IC_50_ = 20 μM). Collectively, analysis of the SAR revealed essential roles of functional groups at both ends of the scaffold for SHP2 inhibitory activity. While the contributions of the benzothiophenone could be reduced to mainly the presence of the carbonyl oxygen (#03), deletion of either the amide group or the hydroxyl group was detrimental to the potency of the inhibitors. Moreover, the nature of the amide *N*-substituent had a significant influence on the activity of analogs.

### Mechanism-of-action and mode of inhibition studies

**Figure 3:**
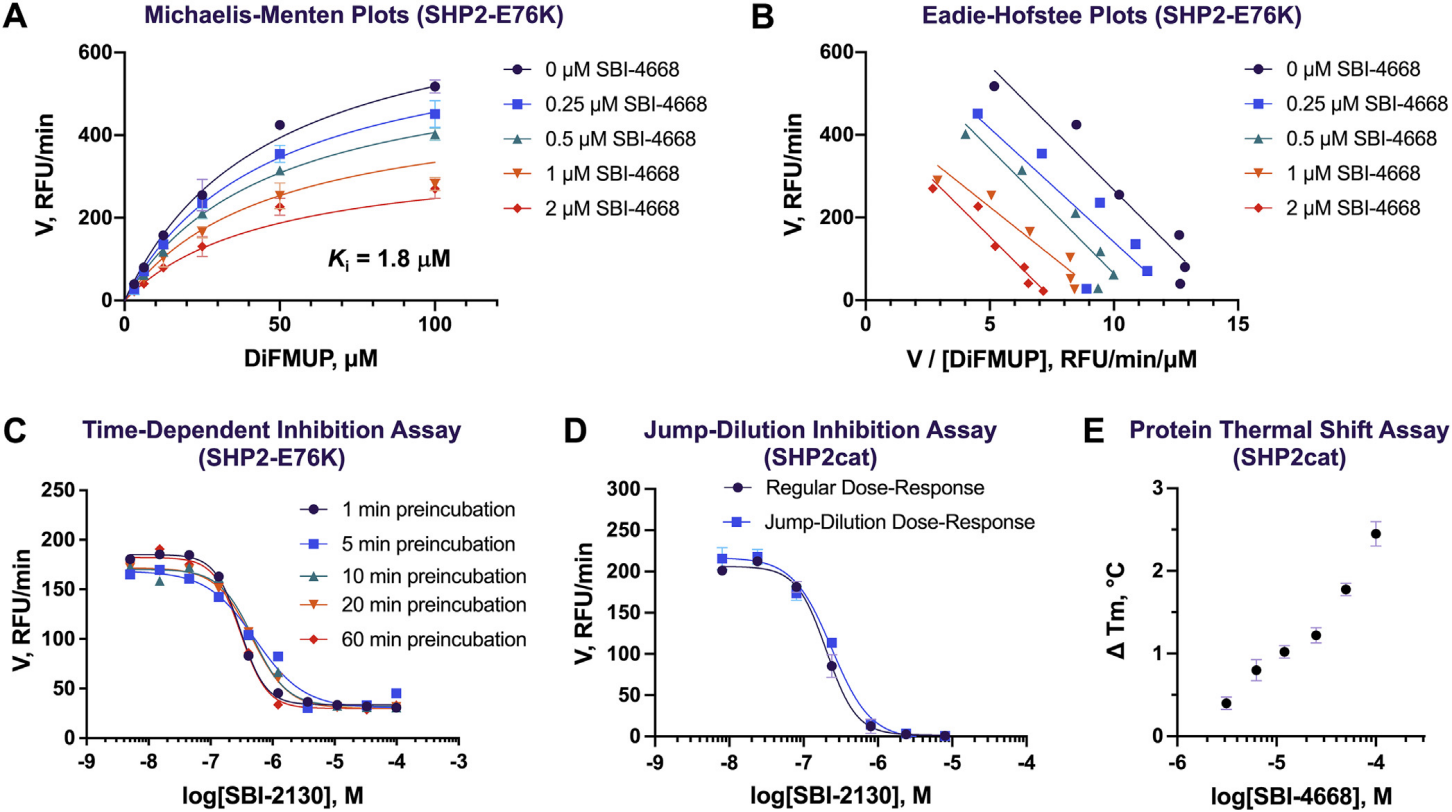
**Mechanism of action, inhibition, and binding studies of SHP2 inhibitors.***A*, Michaelis–Menten kinetic studies for the SHP2 inhibitor SBI-4668 with SHP2-E76K. Plots show the initial rates (V) at various substrates (DiFMUP) and inhibitor concentrations fitted to the Michaelis–Menten equation for noncompetitive inhibition. Relative fluorescence units per minute (RFU/min) are represented as mean ± SD (n = 3). *B*, Eadie–Hofstee plots of the Michaelis–Menten kinetic studies with compound SBI-4668 from *A*. *C*, dose–response curves for SBI-2130 with SHP2-E76K after various preincubation times of inhibitor with SHP2. RFU/min are represented as mean ± SD (n = 3). No time-dependent inhibition was observed as demonstrated by the similar potency for the various time points. *D*, dose–response curves for SBI-2130 with SHP2cat with or without a 10× inhibitor/protein preincubation and jump dilution. Identical IC_50_ curves indicate that SBI-2130 is a reversible inhibitor. RFU/min are represented as mean ± SD (n = 4). *E*, dose-dependent binding of SBI-4668 to SHP2cat in a protein thermal shift (PTS) assay. Thermal stabilization of SHP2 by SBI-4668 is shown by the increase in the SHP2 melting temperature (Δ*T*_m_) compared with vehicle control (DMSO). Δ*T*_m_ values are represented as mean ± SD (n = 4). DiFMUP, 6,8-difluoro-4-methylumbelliferyl phosphate; DMSO, dimethyl sulfoxide; SHP2, Src-homology 2 domain–containing phosphatase 2; SHP2cat, SHP2 catalytic domain.

We previously reported furanylsalicylic acids as potent and selective inhibitors of the *Yersinia* tyrosine phosphatase YopH (37, 38). These prior studies showed that the negatively charged salicylate moiety can act as a pTyr-mimetic and undergoes strong hydrogen bonding interactions with the phosphate-binding loop (P-loop) at the catalytic center, including a salt bridge with the guanidinium group of an invariant arginine that is part of the PTP signature motif (C(X)_5_R) and conserved among all PTPs. Thus, we tested whether replacement of the neutral 2-hydroxybenzamide moiety with the negatively charged salicylate would be beneficial for the potency of this scaffold against SHP2. Interestingly, as mentioned previously, the salicylate analogs, including #06, #07, #09, #10, and #14, were less potent than the *N*-phenyl-2-hydroxybenzamides. These results suggested that the benzamide moiety likely does not act as a pTyr-mimetic or interact with the P-loop residues in SHP2. To corroborate this postulate, we subjected SBI-4668 to Michaelis–Menten kinetic studies with SHP2-E76K to determine its mode of inhibition (Fig. 3, *A* and *B*). Using nonlinear regression, initial rates at various inhibitor and substrate concentrations were fitted to the Michaelis–Menten equations for competitive, noncompetitive, uncompetitive, and mixed inhibitions. Fitting models were then compared using Akaike's Information Criterion (AIC), and inhibition mode probabilities were calculated from the differences between the corresponding second-order corrected AIC scores as previously described (38, 39). The probability data unambiguously indicated that SBI-4668 does not directly compete with substrate binding. Similarly, an uncompetitive inhibition mode was found to be unlikely. Instead, the AIC probability data clearly suggested that SBI-4668 inhibits SHP2 by either a noncompetitive or a mixed inhibition mechanism. The inhibition constant (*K*_*i*_) for noncompetitive inhibition was calculated to be 1.8 μM for SBI-4668, which corresponded well with the measured IC_50_ value.

PTPs contain a highly nucleophilic cysteine that is essential for catalytic activity but is susceptible to oxidation and covalent modifications, which abrogate its nucleophilic function and phosphatase activity (26). Because the methylenebenzothiophenone moiety of our inhibitors could potentially act as a Michael acceptor and thus covalently bind to the catalytic cysteine, we tested whether the observed inhibition of SHP2 was dependent on the preincubation time of inhibitor and enzyme. An irreversible covalent inhibitor may show time-dependent inhibition, with increased potency at longer incubation times. Conversely, the potency of a noncovalent inhibitor is expected to be insensitive to the time it is incubated with the enzyme. To test for time-dependent inhibition, SBI-2130 at ten different concentrations was preincubated with SHP2-E76K for 1, 5, 10, 20, or 60 min, before the DiFMUP substrate was added to the reaction mixture and initial rates were recorded (Fig. 3*C*). The dose–response curves overlapped very well, and IC_50_ values were very similar, suggesting that inhibition of SHP2 by SBI-2130 is not time dependent. Similar results were obtained for SBI-4668 (data not shown). In addition, we used mass spectrometry to test whether inhibitor:protein adducts are being formed. Specifically, we incubated SHP2cat (5 μM) with SBI-4668 (100 μM) in assay buffer for various times (1, 5, 20, and 60 min) before trypsin proteolysis and subsequent mass spectrometry analysis. While the coverage was good, and peptides comprising all existing cysteine residues, including the catalytic cysteine, were detected, no adduct formation with the inhibitor was found (data not shown). Finally, we performed a jump-dilution experiment to test for irreversible inhibition of SHP2. SBI-2130 was preincubated for 10 min with SHP2cat at 10× inhibitor and protein concentrations, compared with a regular dose–response experiment. Preincubation was followed by a 10× jump dilution, incubation for an additional 10 min, and addition of the DiFMUP substrate. An irreversible inhibitor would be expected to shift the IC_50_ curve to lower IC_50_ values in the jump-dilution experiment because of the higher inhibitor concentration during preincubation. In our experiment, in which we tested SBI-2130 in parallel in a jump-dilution and a regular dose–response assay, IC_50_ curves were practically identical (Fig. 3*D*). Collectively, these data suggest that our inhibitors do not act by irreversibly modifying the catalytic cysteine or any other amino acid of SHP2.

### Biophysical binding and inhibitor confirmation in orthogonal assays

We confirmed specific and dose-dependent binding of inhibitors to SHP2 by PTS, which monitors the thermal stability of a target protein *in vitro* (40). Using recombinant SHP2cat in a PTS assay as previously described (36, 41), SBI-4668 dose-dependently increased the melting temperature (*T*_m_) of SHP2cat compared with the vehicle (dimethyl sulfoxide [DMSO]) control (Fig. 3*E*). The data confirm biophysical binding of SBI-4668 to the SHP2 protein within the catalytic domain and agree with the results from the enzyme inhibition assays. In addition, we confirmed the inhibitory activity of several furanylbenzamide inhibitors, including the top two compounds SBI-2130 and SBI-4668, in SHP2cat inhibition assays using the alternative substrate 3-*O*-methylfluorescein phosphate (OMFP), which features a peak fluorescence emission that is red-shifted by ∼75 nm compared with DiFMUP. The *K*_*m*_ value of OMFP for SHP2cat was determined to be 50 μM. An OMFP fluorescence intensity–based assay was used to perform inhibitor dose–response experiments and determine IC_50_ values. Inhibitor potencies were found to be comparable with those determined using DiFMUP. IC_50_ values of SBI-2130 or SBI-4668 against SHP2cat with OMFP were determined to be 0.44 and 1.2 μM, respectively. Collectively, these data demonstrate genuine binding and inhibition of SHP2 by our inhibitors.

### Selectivity of furanylbenzamides for SHP2

Selectivity is one of the biggest challenges in PTP inhibitor development. Therefore, we evaluated potent furanylbenzamides for their ability to selectively inhibit SHP2 over the closely related phosphatases PTP1B and striatal-enriched tyrosine phosphatase (STEP) (Table 2). Similar to SHP2, we adapted a 384-well plate format DiFMUP assay for recombinant PTP1B and STEP and performed Michaelis–Menten experiments to determine DiFMUP *K*_*m*_ values for each phosphatase. IC_50_ values of our inhibitors for PTP1B and STEP were determined in 10-point dose–response assays with DiFMUP used at the concentration corresponding to the respective *K*_*m*_ value. Judging from the IC_50_ values against the catalytic domain of each phosphatase, the most potent SHP2 inhibitor, SBI-2130, also exhibited the best selectivity for SHP2 (31-fold selective over PTP1B; 200-fold selective over STEP). Other inhibitors with good relative selectivity for SHP2 included SBI-4668 (13-fold selective over PTP1B; 73-fold selective over STEP) and SBI-3404 (fivefold selective over PTP1B; >71-fold selective over STEP). Taken together, these results suggest that the furanylbenzamide inhibitors are not pan-active phosphatase inhibitors but instead exhibit a promising level of selectivity for SHP2.

**Table 2:** Selectivity of furanylbenzamide inhibitors for SHP2 over related phosphatases PTP1B and STEP (IC_50_ values in micromolar; each PTP construct was comprised of the catalytic domain)

PTP	SBI-2130	SBI-4668	SBI-3192	SBI-3404	SBI-3405
SHP2	0.22	0.73	1.1	1.4	2.0
PTP1B	6.8	9.4	1.9	7.4	5.3
STEP	44	53	27	>100	7.7

### Efficacy and selectivity of the SHP2 inhibitors in cellular cancer models and patient-derived acute myeloid leukemia samples

**Figure 4:**
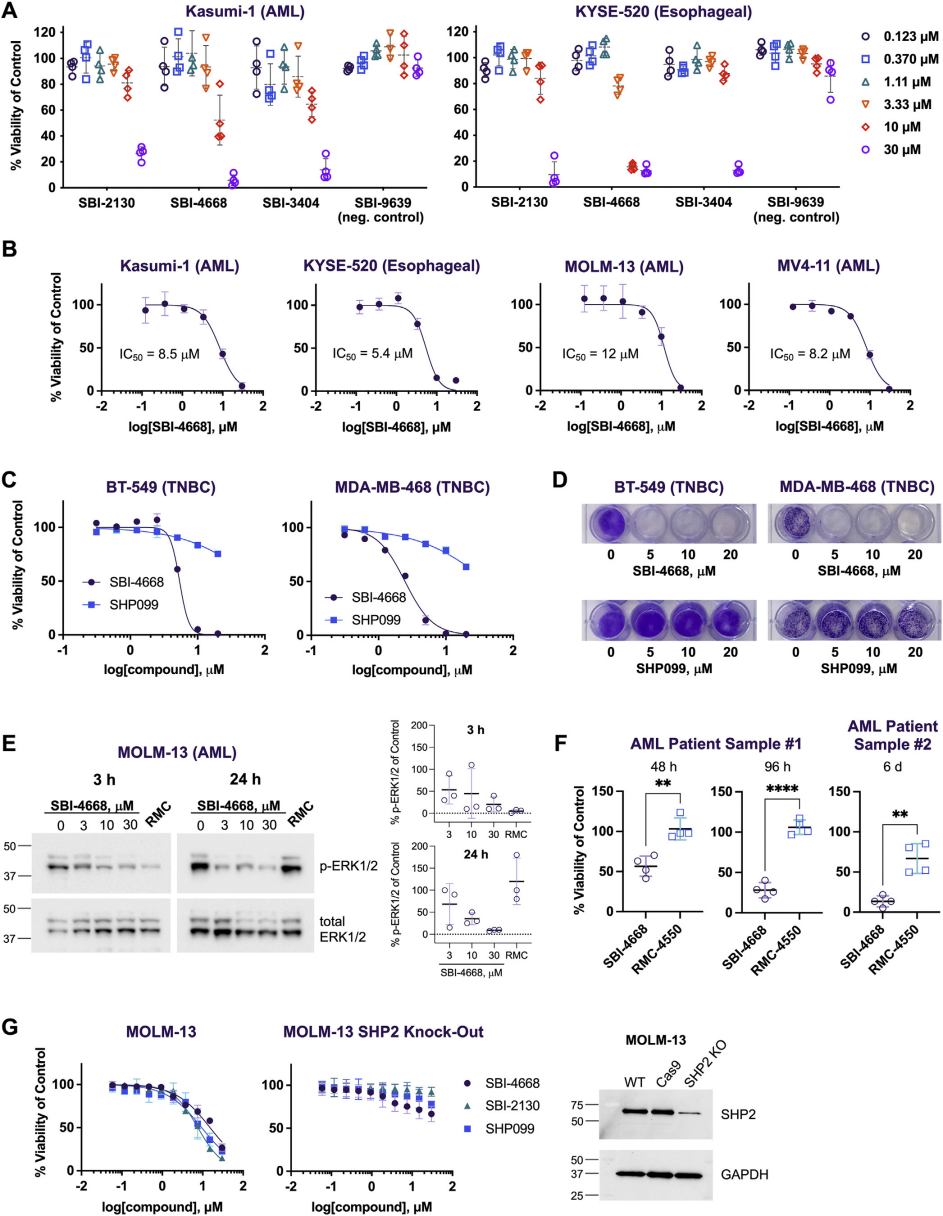
**Efficacy and selectivity of the SHP2 inhibitors in cellular cancer models and in patient-derived AML samples.***A*, viability of AML (Kasumi-1) and esophageal cancer (KYSE-520) cells in the presence of various concentrations of SHP2 active (SBI-2130, SBI-4668, and SBI-3404) and nonactive (SBI6339, negative control) furanylbenzamides after 3 days in culture. Cell viability is shown as a percentage of the vehicle (DMSO) control and represents the mean ± SD (n = 4). *B*, SBI-4668 dose–response curves in cell viability assays using Kasumi-1, KYSE-520, MOLM-13, and MV4-1 cells after 3 days in culture. The percentages compared with DMSO vehicle control were curve fitted using nonlinear regression (log[inhibitor] *versus* normalized response, variable slope) and represent the mean ± SD (n = 4). *C*, cell viability of BT-459 and MDA-MB-468 triple-negative breast cancer (TNBC) cells in the presence of various concentrations of SBI-4668 or the SHP2 allosteric inhibitor SHP099 after 5 days in culture. Cell viability is shown as a percentage of DMSO vehicle control, representing mean ± SD (n = 2), and curve fitted as in *B*. *D*, colony formation assay (11 days) of BT-459 and MDA-MB-468 TNBC cells in the presence of various concentrations of SBI-4668 or the SHP2 allosteric inhibitor SHP099. *E*, phospho-ERK1/2 (p-ERK1/2) immunoblot analysis from total cell lysates of MOLM-13 AML cells treated with SBI-4668 at the indicated concentrations or with SHP2 allosteric inhibitor RMC-4550 (RMC, 1 μM) for 3 h or 24 h. The quantitation of p-ERK1/2 levels is shown as the percentage of the DMSO (vehicle) control and represents data from three independent experiments (mean ± SD). *F*, viability of AML patient-derived cells in the presence of 10 μM SBI-4668 or 10 μM SHP2 allosteric inhibitor RMC-4550 after 2, 4, or 6 days in culture. Cell viability is shown as a percentage of the DMSO vehicle control and represents the mean ± SD (n = 4; ∗*p* < 0.05; ∗∗*p* < 0.01; ∗∗∗∗*p* < 0.0001; unpaired *t* test with Welch's correction). *G*, viability of MOLM-13 cells and MOLM-13-Cas9-mCherry cells with SHP2 KO in the presence of SBI-2130, SBI-4668, and allosteric inhibitor SHP099 at various concentrations (10-point dose response). The percentages compared with the DMSO vehicle control were curve fitted using nonlinear regression (log[inhibitor] *versus* normalized response, variable slope) and represent the mean ± SD (n = 4). SHP2 protein levels in regular MOLM-13 cells (WT), MOLM-13-Cas9-mCherry cells (Cas9), and MOLM-13-Cas9-mCherry cells with SHP2 KO were evaluated by immunoblot analysis using SHP2 antibodies. AML, acute myeloid leukemia; DMSO, dimethyl sulfoxide; SHP2, Src-homology 2 domain–containing phosphatase 2.

Inhibition of SHP2 has been reported to inhibit the growth of cancer cells including Kasumi-1 acute myeloid leukemia (AML) and KYSE-520 esophageal cancer cells (30). Thus, we tested our most potent and selective SHP2 inhibitors (SBI-2130, SBI-4668, and SBI-3404) in cell viability assays using the Kasumi-1 and KYSE-520 cell lines (Fig. 4*A*). We also included an inactive analog (SBI-9639, SHP2 IC_50_ > 100 μM) as a negative control. We found that all three SHP2 inhibitors dose-dependently inhibited cancer cell growth, whereas the inactive analog showed no notable effects. SBI-4668 exhibited the greatest effect on cell growth in both Kasumi-1 cells (IC_50_ = 8.5 μM) and KYSE-520 cells (IC_50_ = 5.4 μM) (Fig. 4*B*). A dose-dependent effect of SBI-4668 on cell viability was also determined in additional AML cell lines, including MOLM-13 (IC_50_ = 12 μM) and MV4-11 (IC_50_ = 8.2 μM). Several studies have revealed that SHP2 is upregulated or hyperactivated in breast cancer, including triple-negative breast cancer (TNBC) (16, 42, 43, 44). Thus, we treated two TNBC cell lines, BT-549 and MDA-MB-468, with SBI-4668 or the SHP2 allosteric inhibitor SHP099 at various concentrations and determined cell viability after 72 h, compared with the vehicle control (Fig. 4*C*). SBI-4668 dose-dependently inhibited the growth of TNBC cells with IC_50_ values of 5.4 μM (BT-549) and 2.5 μM (MDA-MB-468), respectively, whereas SHP099 had a very minor effect on TNBC cell viability. Next, we performed an 11-day colony formation assay with TNBC cells treated with SBI-4668 or SHP099 at 5, 10, or 20 μM (Fig. 4*D*). While vehicle-treated cells retained their capacity to produce colonies, colonies were undetectable for seeded cells treated with SBI-4668. As with the cell viability assays, the SHP2 allosteric inhibitor SHP099 exhibited a very weak effect on colony formation in the TNBC cell lines.

Because SHP2 activity is critical for ERK activation, we used phospho-ERK1/2 (p-ERK1/2) as a more direct readout of SHP2 inhibitor efficacy. MOLM-13 AML cells were treated with either SBI-4668 at 3, 10, or 30 μM or with RMC-4550, a highly potent SHP099-like SHP2 allosteric inhibitor (11), for 3 or 24 h, and p-ERK1/2 levels were detected by immunoblot analysis (Fig. 4*E*). RMC-4550 was used at 1 μM, a concentration that previously was shown to be effective in similar experiments (11). We found that SBI-4668 dose-dependently inhibits ERK1/2 activation in cells treated for either 3 or 24 h. Interestingly, RMC-4550 was highly effective in impeding ERK1/2 activation at the 3 h time point, whereas p-ERK levels in MOLM-13 cells after 24 h treatment were similar to control cells treated with vehicle. This p-ERK rebound in MOLM-13 cells treated with RMC-4550 is reminiscent of previously reported data from human hepatoma Hep3B cells treated with SHP099 (34). We also tested the effects of SBI-4668 or RMC-4550 on two patient-derived AML samples (Fig. 4*F*). SBI-4668 at 10 μM inhibited the growth of both AML patient samples, whereas RMC-4550 at 10 μM only affected the growth of sample 2, albeit to a lesser extent than SBI-4668. Collectively, these data demonstrate the efficacy of our inhibitors in various cell culture models of cancer, including in primary patient-derived cells.

Finally, we evaluated the selectivity of our top two compounds, SBI-2130 and SBI-4668, on MOLM-13 AML cells in which SHP2 was depleted using CRISPR–Cas9 KO. High-efficiency Cas9-editing MOLM-13 cells were generated by transducing MOLM-13 cells with a Cas9 lentiviral construct. Stable clones were tested for editing efficiency by performing tracking of indels by decomposition analysis (45), and these MOLM-13-Cas9 cells were transduced with a lentiviral construct containing an *AAVS1* single-guide RNA (sgRNA) and an mCherry reporter for bulk sorting of cells with successful *AAVS1* editing. SHP2 KO was achieved by transducing MOLM-13-Cas9-mCherry cells with a lentivirus, containing SHP2 dual sgRNAs, resulting in ∼80% reduction of SHP2 protein levels (Fig. 4*G*). SBI-2130, SBI-4668, and the SHP2 allosteric inhibitor, SHP099, were tested in parallel on regular MOLM-13 cells (expressing WT SHP2) and MOLM-13-Cas9-mCherry cells with SHP2 KO. Cells were treated with inhibitors at various concentrations (ranging from 30 μM to 58 nM) or vehicle control (DMSO) for 72 h, before cell viability was assessed. All three SHP2 inhibitors inhibited the cell viability of MOLM-13 cells with IC_50_ values of 15 (SBI-4668), 7.6 (SBI-2130), and 9.4 μM (SHP099). In contrast, the inhibitors had either no effect, or a greatly reduced effect, on the viability of MOLM-13-Cas9-mCherry SHP2 KO cells, demonstrating the selectivity of the compounds for SHP2 under physiological conditions (Fig. 4*G*). In summary, the data demonstrate both efficacy and selectivity of our inhibitors in cancer cells expressing WT SHP2.

### Evaluation of SBI-4668 in oncogenic mutant SHP2 cellular models

**Figure 5:**
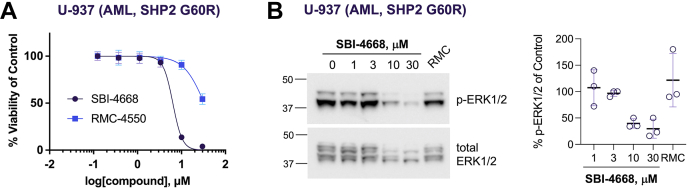
**Evaluation of SBI-4668 in U-937 AML cells expressing the SHP2 oncogenic variant G60R.***A*, cell viability of U-937 AML cells in the presence of various concentrations of SBI-4668 or the SHP2 allosteric inhibitor RMC-4550 after 3 days in culture. Cell viability is shown as a percentage of the DMSO vehicle control, representing the mean ± SD (n = 4) and curve fitted using nonlinear regression (log[inhibitor] *versus* normalized response, variable slope). *B*, phospho-ERK1/2 (p-ERK1/2) immunoblot analysis from total cell lysates of U-937 AML cells treated with SBI-4668 at the indicated concentrations or with SHP2 allosteric inhibitor RMC-4550 (RMC, 1 μM) for 24 h. The quantitation of p-ERK1/2 levels is shown as the percentage of the DMSO (vehicle) control and represents data from three independent experiments (mean ± SD). AML, acute myeloid leukemia; DMSO, dimethyl sulfoxide; SHP2, Src-homology 2 dom/ain–containing phosphatase 2.

Oncogenic gain-of-function mutations in SHP2 drive leukemogenesis in a significant number of leukemia patients. However, because of their unique mechanism of action, the existing SHP2 allosteric inhibitors lack activity against the most common SHP2 oncogenic variants. Thus, we tested whether our inhibitors could inhibit the growth of leukemia cells expressing oncogenic SHP2 variants. Importantly, we previously established the direct target engagement of SBI-4668 with mutant SHP2 in live cells using a cellular PTS assay (cellular thermal shift assay) (36, 41). We also previously found that the potent SHP2 allosteric inhibitor RMC-4550 shows a greatly decreased cellular target engagement with the SHP2 E76K mutant compared with WT SHP2 (36, 41). Thus, we tested both SBI-4668 and RMC-4550 on U-937 AML cells, which harbor a G60R oncogenic mutation in SHP2. We treated cells with either SBI-4668 or RMC-4550 at various concentrations, ranging from 0.123 to 30 μM (Fig. 5*A*). In agreement with prior reports (31, 32, 46), the SHP099-like allosteric inhibitor RMC-4550 had a very weak effect on the viability of cells expressing the SHP2 gain-of-function variant (IC_50_ = 33 μM). By contrast, SBI-4668 inhibited U-937 cell growth with an IC_50_ value of 6.3 μM, which is comparable to the potency found in AML cells expressing WT SHP2. We also assessed the effects of SBI-4668 and RMC-4550 on ERK1/2 activation in the SHP2 mutant U-937 cell line. Similar to the immunoblot experiments described for MOLM-13 cells above, U-937 AML cells were treated with either vehicle control (DMSO), SBI-4668 (1, 3, 10, or 30 μM), or with RMC-4550 (1 μM) for 24 h and processed for p-ERK1/2 immunoblot analysis (Fig. 5*B*). While SBI-4668 dose-dependently inhibited ERK1/2 activation, a decrease in p-ERK1/2 levels was not detectable after treatment with RMC-4550. Collectively, our data demonstrate similar efficacy of our inhibitor on AML cells expressing a common SHP2 oncogenic variant compared with AML cells expressing WT SHP2.

## Discussion

SHP2 has become an attractive therapeutic target for cancers with upregulated RTK and Ras signaling. Several compounds that allosterically inhibit SHP2 are in phase I/II clinical trials, either as a monotherapy or in combination with other RTK/Ras pathway and immune checkpoint inhibitors. The new class of SHP2 allosteric inhibitors may ultimately prove to be transformative for the treatment of many cancers. However, because of the unique mechanism of action, they will likely be ineffective for patients with cancers that are driven by oncogenic mutant forms of SHP2, most notably pediatric and acute leukemias. Among all blood cancers, the highest rates of SHP2 gain-of-function mutations occur in juvenile myelomonocytic leukemia, with leukemogenesis driven by SHP2 variants in up to 42% of these children (source: COSMIC database, March 2021). Acute leukemias with larger patient populations, such as AML and B-cell acute lymphoblastic leukemia, have SHP2 mutation rates between 5 and 10%, and those mutations are associated with poor clinical outcomes as well as resistance to targeted therapies (47, 48). Finally, cancers driven by aberrant FGF signaling, such as many breast cancers, seem to be inherently resistant to current SHP2 allosteric inhibitors (34). The reason for this resistance is a rapid feedback activation of the FGFR that leads to increased recruitment and activation of SHP2. Clearly, there is an urgent need for novel SHP2 inhibitory compounds that act through different mechanisms and exhibit a new inhibition paradigm.

Here, we report the synthesis and SAR around a series of furanylbenzamides that bind and inhibit oncogenic and WT forms of SHP2. To characterize these compounds, we have adopted a comprehensive biophysical, biochemical, and cellular testing platform. Several inhibitors exhibited substantial responses in various cancer cellular models, including TNBC and AML. Importantly, and in contrast to existing SHP2 allosteric inhibitors, our compounds effectively inhibit the growth of AML cells expressing a frequent SHP2 oncogenic variant. Moreover, we found a prolonged cellular response caused by SBI-4668 treatment, compared with the allosteric inhibitor RMC-4550, based on the compounds' inhibitory effect on ERK1/2 pathway activation. Target engagement of SBI-4668 with SHP2 was confirmed *in vitro* using PTS and in cells using a cellular thermal shift assay as previously reported (36). Selectivity of the inhibitors for SHP2 was established *in vitro* against two related phosphatases and in cells using CRISPR–Cas9 to knockout SHP2. Compared with regular MOLM-13 cells, efficacy of SBI-2130 and SBI-4668 was either completely abrogated or greatly reduced in MOLM-13-Cas9-mCherry cells in which SHP2 levels had been greatly reduced, suggesting that on-target activity was the primary cause of cytotoxicity caused by the inhibitors. Using both biochemical and biophysical assessments, we could pinpoint the interaction of furanylbenzamide inhibitors with the phosphatase domain of SHP2. A possible covalent mechanism *via* Michael addition to the enone double bond common to this series of compounds could be excluded, based on time-dependent inhibition, jump-dilution inhibition, and mass spectroscopic analyses. Interestingly, Michaelis–Menten kinetic experiments suggested that our compounds do not directly compete with substrate binding at the active site. This notion was further supported by SAR studies, in which analogs with increased substrate resemblance exhibited a decrease in potency, suggesting that SBI-2130, SBI-4668, and related analogs likely do not bind to the phosphate-binding loop in the catalytic pocket. Future studies will be necessary to determine the exact binding site of the compounds with SHP2. Such studies will also enable a structure-based rational design of additional analogs with further improved potency and selectivity.

## Experimental procedures

### Chemistry and synthetic procedures

All reactions were performed in oven-dried glassware under an atmosphere of nitrogen with magnetic stirring. All solvents and chemicals used were purchased from Sigma–Aldrich or Acros and used as received. Purity and characterization of compounds were established by a combination of LC–MS and NMR analytical techniques and was >95% for all tested compounds. Silica gel column chromatography was carried out using prepacked silica cartridges from RediSep (ISCO Ltd) and eluted using an Isco Companion system. ^1^H-NMR and ^13^C-NMR spectra were obtained on a JEOL 400 spectrometer at 400 and 100 MHz, respectively. Chemical shifts are reported in *δ* (ppm) relative to residual solvent peaks or tetramethylsilane as internal standards. J-coupling constants are reported in hertz. High-resolution electrospray ionization (ESI)–TOF mass spectra were acquired from the Mass Spectrometry Core at Sanford Burnham Prebys Medical Discovery Institute. HPLC–MS analyses were performed on a Shimadzu 2010EV LCMS using the following conditions: Kromisil C18 column (reverse phase, 4.6 × 50 mm); a linear gradient from 10% acetonitrile and 90% water to 95% acetonitrile and 5% water over 4.5 min; flow rate of 1 ml/min; and UV photodiode array detection from 200 to 300 nm.

### (Z)-2-((5-Bromofuran-2-yl)methylene)benzo[b]thiophen-3(2H)-one (intermediate III)

To a vigorously stirred solution of lithium diisopropylamide (22 mmol) in anhydrous tetrahydrofuran (THF) (25 ml) at 0 °C, a solution of *N,N*-diethyl-2-(methylthio)benzamide (2.23 g, 10 mmol) and 5-bromo-2-furaldehyde (1.74 g, 10 mmol) in anhydrous THF (5 ml) was added under nitrogen atmosphere. The resulting mixture was stirred for 2 h and then gradually warmed to room temperature (RT). The reaction mixture was then poured into water. The pH was adjusted to 4 to 5 by addition of diluted hydrochloric acid. The organic layer was separated, and the aqueous layer was extracted with ethyl acetate (EtOAc). The organic phases were combined and dried over anhydrous Na_2_SO_4_. Evaporation of the solvent followed by silica gel chromatography using hexanes/EtOAc (20:1) yielded (Z)-2-((5-bromofuran-2-yl)methylene)benzo[b]thiophen-3(2H)-one III. *Orange solid* (2 g, 65.7%). ^1^H NMR (400 MHz, CDCl_3_): *δ* 7.88 (d, *J* = 7.8 Hz, 1H), 7.61 (s, 1H), 7.55 (t, *J* = 7.8 Hz, 1H), 7.48 (d, *J* = 7.8 Hz, 1H), 7.28 (t, *J* = 7.3 Hz, 1H), 6.78 (d, *J* = 3.7 Hz, 1H), 6.50 (d, *J* = 3.7 Hz, 1H). ^13^C NMR (101 MHz, CDCl_3_): *δ* 188.2, 152.9, 146.1, 135.2, 130.6, 128.9, 126.9, 125.5, 123.9, 119.5, 117.9, 115.3. LC–MS (ESI) [M + H]^+^: 307.

### 2-Hydroxy-5-(5-((2-oxocyclopentylidene)methyl)furan-2-yl)-*N*-phenylbenzamide (#03, SBI-3192)

To a stirred solution of 2-hydroxy-5-(5-((2-oxocyclopentylidene)methyl)furan-2-yl)benzoic acid (0.050 g, 0.17 mmol) in *N*,*N*-dimethylformamide (DMF) (3 ml) was added aniline (0.023 g, 025 mmol), *N*,*N*-diisopropylethylamine (DIEA) (0.088 ml, 0.50 mmol), and chlorodipyrrolidinocarbenium hexafluorophosphate (PyClU) (0.084 g, 0.25 mmol) at RT. The resulting mixture was stirred for 2 h. The reaction mixture was diluted with EtOAc and quenched with the addition of saturated NH_4_Cl. The layers were separated, and the aqueous layer was washed with EtOAc for three times. The combined organic layer was dried over anhydrous Na_2_SO_4_, concentrated to give the crude product, which was purified by reverse-phase HPLC. *Dark red solid* (0.033 g, 53%). ^1^H NMR (400 MHz, DMSO-d_6_): *δ* 8.29 (t, *J* = 2.7 Hz, 1H), 7.83 (dd, *J* = 8.9, 2.5 Hz, 1H), 7.75 to 7.69 (m, 2H), 7.66 (d, *J* = 8.0 Hz, 1H), 7.43 to 7.35 (m, 2H), 7.14 (t, *J* = 6.9 Hz, 1H), 7.09 to 7.00 (m, 4H), 3.04 (dt, *J* = 8.1, 4.2 Hz, 2H), 2.03 to 1.97 (m, 2H), 1.96 to 1.87 (m, 2H). LC–MS (ESI) [M + H]^+^: 374.

### (Z)-2-((5-(4-Hydroxy-3-(2H-tetrazol-5-yl)phenyl)furan-2-yl)methylene)benzo[b]thiophen-3(2H)-one (#04, SBI-3405)

(Z)-2-Hydroxy-5-(5-((3-oxobenzo[b]thiophen-2(3H)-ylidene)methyl)furan-2-yl)benzonitrile (0.070 g, 0.2 mmol), sodium azide (0.156 g, 2.4 mmol), and ammonium chloride (0.127 g, 2.4 mmol) were taken in DMF (4 ml), and the resultant mixture was heated at 100 °C for 1 h. The reaction mixture was cooled to RT and diluted with water and extracted with EtOAc (3 × 10 ml). The organic phases were combined and dried over anhydrous Na_2_SO_4_. Evaporation of the solvent followed by reverse-phase HPLC yielded title compound. *Dark red solid* (0.049 g, 64%). ^1^H NMR (400 MHz, DMSO-d_6_): *δ* 8.52 (d, *J* = 2.3 Hz, 1H), 7.99 to 7.95 (m, 1H), 7.85 (d, *J* = 7.8 Hz, 1H), 7.80 (d, *J* = 7.8 Hz, 1H), 7.79 (s, 1H), 7.72 (t, *J* = 8.2 Hz, 1H), 7.39 (t, *J* = 7.8 Hz, 1H), 7.35 (d, *J* = 3.7 Hz, 1H), 7.27 (d, *J* = 3.7 Hz, 1H), 7.24 (d, *J* = 7.8 Hz, 1H). LC–MS (ESI) [M + H]^+^: 389.

### (Z)-*N*-(3-Aminophenyl)-2-hydroxy-5-(5-((3-oxobenzo[b]thiophen-2(3H)-ylidene)methyl)furan-2-yl)benzamide (#08, SBI-2126)

(Z)-2-Hydroxy-5-(5-((3-oxobenzo[b]thiophen-2(3H)-ylidene)methyl)furan-2-yl)benzoic acid (0.05 g, 0.137 mmol) and 1,1′-carbonyldiimidazole (0.022 g, 0.137 mmol) were taken in DMF (2 ml) and stirred for 1 h. To this, benzene-1,3-diamine (0.030 g, 0.274 mmol) was added, and stirring was continued for another 1 to 2 h. After complete consumption of the starting material, the reaction mixture was diluted with water and extracted with EtOAc. The combined organic extracts were washed with water, dried over anhydrous Na_2_SO_4_, and concentrated under reduced pressure to yield the crude product, which was further purified by reverse-phase HPLC. *Red solid* (0.036 g, 58%). ^1^H-NMR (400 MHz, DMSO-d_6_): δ 10.23 (s, 1H), 8.23 (1H), 7.61 to 8.03 (9H), 7.24 to 7.40 (3H), 7.14 (s, 1H), 6.95 (d, *J* = 8.2 Hz, 1H). ^13^C-NMR (101 MHz, DMSO-d_6_): δ 187.4, 163.3, 160.1, 157.7, 157.2, 149.9, 145.9, 145.8, 136.0, 131.6, 131.5, 129.7, 127.0, 126.8, 126.6, 126.4, 126.2, 125.1, 124.9, 123.6, 123.4, 119.9, 109.5. LC–MS (ESI) [M + H]^+^: 455.

### (Z)-2-((5-(4-Hydroxyphenyl)furan-2-yl)methylene)benzo[b]thiophen-3(2H)-one (#17, SBI-4167)

To a mixture of 2-((5-bromofuran-2-yl)methylene)benzo[b]thiophen-3(2H)-one (0.307 g, 1 mmol), (4-hydroxyphenylboronic acid (0.206 g, 1.5 mmol)), and tetrakistriphenylphosphinepalladium(0) (0.115 g, 0.1 mmol) in dimethoxyethane (5 ml) was added a 2 M Na_2_CO_3_ solution (0.5 ml). The resultant solution was heated at reflux in an atmosphere of nitrogen for 12 h. The reaction mixture was cooled to RT and diluted with water and then acidified using 1 N HCl. The aqueous phase was extracted with EtOAc (3 × 10 ml), and the combined organic layer was washed with brine, followed by drying over anhydrous Na_2_SO_4_. Filtration and removal of the solvent-afforded crude product, which was further purified by automated prep-HPLC to yield the desired compound. *Dark red solid* (0.236 g, 73.7%). ^1^H NMR (400 MHz, DMSO-d_6_): *δ* 10.03 (s, 1H), 7.86 to 7.81 (m, 2H), 7.77 (d, *J* = 8.3 Hz, 3H), 7.70 to 7.68 (m, 1H), 7.42 to 7.35 (m, 1H), 7.33 to 7.30 (m, 1H), 7.18 (d, *J* = 3.6 Hz, 1H), 6.90 (d, *J* = 8.7 Hz, 2H). ^13^C NMR (101 MHz, DMSO-d_6_): *δ* 187.4, 159.2, 158.7, 149.4, 145.9, 135.8, 130.9, 126.8, 126.7, 126.3, 126.2, 126.1, 125.0, 123.6, 120.6, 119.3, 116.6, 108.5. LC–MS (ESI) [M + H]^+^: 320.95.

### 5-(Furan-2-yl)-2-hydroxy-*N*-phenylbenzamide (#21, SBI-4232)

To a solution of 5-bromo-2-hydroxybenzoic acid (0.434 g, 1 mmol) in DMF (10 ml) was added hydroxybenzotriazole (HOBt) (0.459 g, 3 mmol) and 1-ethyl-3-(3-dimethylaminopropyl)carbodiimide (EDC) (0.575 g, 3 mmol), and the resulting mixture was stirred at for 30 min. To this mixture, aniline (0.279 g, 3 mmol) and DIEA (1.05 ml, 6 mmol) were added. The resulting mixture was stirred for 2 h. The reaction mixture was diluted with EtOAc and quenched with the addition of saturated NH_4_Cl. The layers were separated, and the aqueous layer was washed three times with EtOAc. The combined organic layer was dried over anhydrous Na_2_SO_4_, concentrated to give the crude product as a light-yellow solid, which was used for next step without further purifications. *White solid* (0.569 g, 97.4%). LC–MS (ESI) [M + H]^+^: 292.95. A mixture of 5-bromo-2-hydroxy-*N*-phenylbenzamide (0.569 g, 1.948 mmol), 2-furanboronic acid 7 (0.326 g, 2.92 mmol), and (Ph_3_P)_4_Pd (0.0225 g, 0.0.018 mmol) in dioxane (25 ml) and 2 M aqueous Na_2_CO_3_ (3.9 ml) was flushed with nitrogen for 5 min and heated at 80 °C for 12 h under nitrogen atmosphere. The solvents were removed under reduced pressure, the residue was dissolved in water (1000 ml), the mixture obtained was filtered through Celite, and the filtrate was neutralized with 2 N hydrochloric acid. The solids were filtered, washed with water, dried, and recrystallized from ethanol to give 5-(furan-2-yl)-2-hydroxybenzoic acid. *Pale yellow solid* (0.350 g, 64.3%). ^1^H NMR (400 MHz, DMSO-d_6_): *δ* 11.87 (s, 1H), 10.49 (s, 1H), 8.25 (d, *J* = 2.2 Hz, 1H), 7.80 to 7.66 (m, 4H), 7.39 (t, *J* = 7.6 Hz, 2H), 7.15 (t, *J* = 7.5 Hz, 1H), 7.05 (dd, *J* = 8.6, 1.7 Hz, 1H), 6.84 (d, *J* = 3.1 Hz, 1H), 6.62 to 6.53 (m, 1H). ^13^C NMR (101 MHz, DMSO-d_6_): *δ* 166.1, 157.6, 152.6, 142.32, 138.1, 128.8, 124.3, 124.2, 122.1, 122.0, 121.0, 120.8, 118.3, 117.8, 112.0, 104.5. LC–MS (ESI) [M + H]^+^: 279.95.

### (Z)-2-((5-phenylfuran-2-yl)methylene)benzo[b]thiophen-3(2H)-one (#25, SBI-3570)

To a mixture of 2-((5-bromofuran-2-yl)methylene)benzo[b]thiophen-3(2H)-one (0.077 g, 0.25 mmol), phenylboronic acid (0.0.37 g, 0.3 mmol), and tetrakistriphenylphosphinepalladium(0) (0.029 g, 0.025 mmol) in dimethoxyethane (5 ml) was added a 2 M Na_2_CO_3_ solution (0.5 ml). The resulting solution was heated at reflux in an atmosphere of nitrogen for 6 to 12 h. The reaction mixture was cooled to RT and diluted with water and then acidified using 1 N HCl. The aqueous phase was extracted with EtOAc (3 × 10 ml), and the combined organic layer was washed with brine, followed by drying over anhydrous Na_2_SO_4_. Filtration and removal of the solvent-afforded crude product, which was further purified by automated prep-HPLC to yield the desired compound. *Orange solid* (0.063 g, 83%). ^1^H NMR (400 MHz, CDCl_3_): *δ* 7.90 (d, *J* = 8.7 Hz, 1H), 7.83 (d, *J* = 9.1 Hz, 2H), 7.73 (s, 1H), 7.56 to 7.53 (m, 2H), 7.47 (t, *J* = 7.8 Hz, 2H), 7.36 (t, *J* = 7.8 Hz, 1H), 7.28 to 7.26 (m, 1H), 6.94 (d, *J* = 3.7 Hz, 1H), 6.85 (d, *J* = 3.7 Hz, 1H). ^13^C NMR (101 MHz, CDCl_3_): *δ* 188.2, 157.6, 150.3, 146.4, 134.8, 130.9, 129.4, 128.9, 128.8, 127.9, 126.8, 125.3, 124.6, 123.9, 120.7, 118.9, 108.9. LC–MS (ESI) [M + H]^+^: 305.

### (Z)-2-Hydroxy-5-(5-((3-oxobenzofuran-2(3H)-ylidene)methyl)furan-2-yl)benzoic acid (#07, SBI-0165): method A

To an ice-cooled solution of benzofuranone (0.134 g, 1 mmol) in EtOH (8 ml) was added a solution of NaOH (0.200 g, 5 mmol) in 2 ml water dropwise. To this, 5-(5-formylfuran-2-yl)-2-hydroxybenzoic acid (0.116 g, 0.5 mmol) was added, and the resulting mixture was gradually warmed to RT and stirred for another 1 h. Reaction mixture was then diluted with water and acidified using 1 N HCl. The precipitated product was collected by filtration and washed with water and dried to yield the product as a *dark red solid* (0.244 g, 70%). Because of the low solubility of the product, only a few milligrams of the crude material were purified by reverse-phase HPLC to yield pure material for the biochemical assays. ^1^H-NMR (400 MHz, DMSO-d_6_): δ 8.23 (d, *J* = 2.3 Hz, 1H), 7.96 (dd, *J* = 8.7, 2.3 Hz, 1H), 7.76 to 7.73 (m, 2H), 7.47 (d, *J* = 8.2 Hz, 1H), 7.30 to 7.26 (m, 3H), 7.18 (d, *J* = 3.7 Hz, 1H), 7.06 (d, *J* = 8.7 Hz, 1H), 6.92 (s, 1H). ^13^C-NMR (101 MHz, DMSO-d_6_): δ 183.0, 171.9, 165.4, 161.9, 156.0, 147.9, 144.7, 137.7, 132.0, 126.6, 124.7, 124.4, 122.0, 121.4, 118.7, 113.6, 109.2, 101.4. LC–MS (ESI) [M + H]^+^: 349.

### 2-Hydroxy-5-(5-((2-oxocyclohexylidene)methyl)furan-2-yl)benzoic acid (#14, SBI-3194)

This compound was prepared according to method A using cyclohexanone (0.098 g, 1 mmol), NaOH (0.200 g, 5 mmol), and 5-(5-formylfuran-2-yl)-2-hydroxybenzoic acid (0.116 g, 0.5 mmol). *Dark red solid* (0.126 g, 80.7%). Because of the low solubility of the product, only few milligrams were purified by reverse-phase HPLC, yielding pure compound for testing. ^1^H NMR (400 MHz, DMSO-d_6_): *δ* 8.16 (d, *J* = 2.3 Hz, 1H), 7.93 (dd, *J* = 8.7, 2.4 Hz, 1H), 7.27 (t, *J* = 2.3 Hz, 1H), 7.12 (d, *J* = 3.6 Hz, 1H), 7.08 (d, *J* = 8.7 Hz, 1H), 7.02 (d, *J* = 3.6 Hz, 1H), 2.98 to 2.88 (m, 2H), 2.43 (t, *J* = 6.3 Hz, 2H), 1.90 to 1.74 (m, 4H). LC–MS (ESI) [M + H]^+^: 313.

### 2-Hydroxy-5-(5-((1-oxo-1,3-dihydro-2H-inden-2-ylidene)methyl)furan-2-yl)benzoic acid (#09, SBI-3204)

This compound was prepared according to method A using 2,3-dihydro-1H-inden-1-one (0.066 g, 0.5 mmol), NaOH (0.080 g, 2 mmol), and 5-(5-formylfuran-2-yl)-2-hydroxybenzoic acid (0.116 g, 0.5 mmol). *Red* (0.154 g, 93.2%). Because of the low solubility of the product, only few milligrams were purified by reverse-phase HPLC, yielding pure compound for testing. Yield represents crude material yield. ^1^H-NMR (400 MHz, DMSO-d_6_): δ 8.26 (d, *J* = 2.4 Hz, 1H), 8.04 (dd, *J* = 8.7, 2.3 Hz, 1H), 7.95 (s, 1H), 7.77 (d, *J* = 7.6 Hz, 1H), 7.75 to 7.65 (m, 3H), 7.49 (td, *J* = 7.5, 1.3 Hz, 1H), 7.40 (t, *J* = 2.0 Hz, 1H), 7.21 (q, *J* = 3.7 Hz, 2H), 7.12 (d, *J* = 8.7 Hz, 1H), 4.15 (d, *J* = 2.0 Hz, 2H). LC–MS (ESI) [M + H]^+^: 331.

### (Z)-2-Hydroxy-5-(5-((3-oxobenzo[b]thiophen-2(3H)-ylidene)methyl)thiophen-3-yl)benzoic acid (#10, SBI-6999): Method B

To a vigorously stirred solution of lithium diisopropylamide (4 mmol) in anhydrous THF (5 ml) at 0 °C, a solution of *N*,*N*-diethyl-2-(methylthio)benzamide (0.223 g, 1 mmol) and 5-(5-formylfuran-2-yl)-2-hydroxybenzoic acid (0.232 g, 1 mmol) in anhydrous THF (5 ml) was added under nitrogen atmosphere. The resulting mixture was stirred for 2 h and then gradually warmed to RT. The reaction mixture was then poured into water. The pH was adjusted to 4 to 5 by addition of diluted HCl. The organic layer was separated, and the aqueous layer was extracted with EtOAc (3 × 10 ml). The organic phases were combined and dried over anhydrous Na_2_SO_4_. Evaporation of the solvent followed by reverse-phase HPLC yielded (Z)-2-hydroxy-5-(5-((3-oxobenzo[b]thiophen-2(3H)-ylidene)methyl)thiophen-3-yl)benzoic acid. *Red solid* (0.222 g, 61%). ^1^H NMR (400 MHz, DMSO-d_6_): *δ* 8.30 (d, *J* = 2.3 Hz, 1H), 8.02 (dd, *J* = 2.3 Hz, 8.7 Hz, 1H), 7.83 (d, *J* = 7.8 Hz, 1H), 7.74 (s, 1H), 7.72 to 7.68 (m, 2H), 7.38 (t, *J* = 7.8 Hz, 1H), 7.31 (d, *J* = 3.7 Hz, 1H), 7.24 (d, *J* = 3.7 Hz, 1H), 7.13 (d, *J* = 8.7 Hz, 1H). ^13^C NMR (101 MHz, DMSO-d_6_): *δ* 186.9, 171.3, 161.5, 156.5, 149.4, 145.3, 135.5, 131.3, 130.3, 126.5, 126.3, 126.2, 125.9, 124.4, 122.8, 120.5, 118.8, 118.3, 113.9, 109.1. LC–MS (ESI) [M + H]^+^: 365. High-resolution MS (ESI) calculated for C_20_H_12_O_5_S [M + H]^+^: 365.0417. Found: 365.0481.

### (Z)-2-Hydroxy-5-(5-((3-oxobenzo[b]thiophen-2(3H)-ylidene)methyl)furan-2-yl)benzonitrile (#05, SBI-3404)

This compound was prepared according to method B using *N*,*N*-diethyl-2-(methylthio)benzamide (0.223 g, 1 mmol) and 5-(5-formylfuran-2-yl)-2-hydroxybenzonitrile (0.214 g, 1 mmol). *Red solid* (0.210 g, 61%). ^1^H NMR (400 MHz, DMSO-d_6_): *δ* 11.68 (brs, 1H), 8.13 (d, *J* = 1.8 Hz, 1H), 8.01 (dd, *J* = 2.3 Hz, 8.7 Hz, 1H), 7.83 to 7.81 (m, 2H), 7.79 (s, 1H), 7.71 (t, *J* = 7.3 Hz, 1H), 7.38 (t, *J* = 7.8 Hz, 1H), 7.31 (d, *J* = 3.7 Hz, 1H), 7.26 (d, *J* = 3.7 Hz, 1H), 7.18 (d, *J* = 8.7 Hz, 1H). LC–MS (ESI) [M + H]^+^: 346.

### 2-Hydroxy-5-(5-((2-oxocyclopentylidene)methyl)furan-2-yl)benzoic acid (#06, SBI-3191)

This compound was prepared according to method B using cyclopentanone (0.084 g, 1 mmol), NaOH (0.200 g, 5 mmol), and 5-(5-formylfuran-2-yl)-2-hydroxybenzoic acid (0.116 g, 0.5 mmol). *Dark red solid* (0.110 g, 73.8%). ^1^H NMR (400 MHz, DMSO-d_6_): *δ* 8.13 (dd, *J* = 4.1, 2.3 Hz, 1H), 7.92 to 7.88 (m, 1H), 7.86 (dd, *J* = 6.4, 3.0 Hz, 1H), 7.09 (d, *J* = 3.7 Hz, 1H), 7.04 (d, *J* = 8.7 Hz, 1H), 7.01 (dd, *J* = 5.6, 3.3 Hz, 2H), 6.76 (s, 0H), 2.81 to 2.73 (m, 2H), 2.00 to 1.92 (m, 2H), 1.92 to 1.82 (m, 2H). LC–MS (ESI) [M + H]^+^: 299.

### General procedure for the synthesis of amide derivatives: Method C

To a stirred solution of acid (0.2 mmol, 1 equivalent) in DMF (2 ml) at RT was added HOBt (0.24 mmol, 1.2 equivalent) in one portion followed by EDC (0.24 mmol, 1.2 equivalent). The resulting mixture was stirred at RT for 30 min. To this mixture, amine (0.24 mmol, 1.2 equivalent) and DIEA (0.24 mmol, 1.2 equivalent) were added and stirred for 2 h. After complete consumption of the starting material, the reaction mixture was diluted with water and extracted with EtOAc. The combined organic extracts were washed with water, dried over anhydrous Na_2_SO_4_, and concentrated under reduced pressure to yield the crude product that was further purified by reverse-phase HPLC.

### (Z)-*N*-(4-Aminophenyl)-2-hydroxy-5-(5-((3-oxobenzo[b]thiophen-2(3H)-ylidene)methyl)furan-2-yl)benzamide (#01, SBI-2130)

This compound was prepared according to method C. *Red solid* (0.053 g, 58%). ^1^H NMR (400 MHz, DMSO-d_6_): *δ* 10.22 (s, 1H), 8.41 (s, 1H), 7.94 (d, *J* = 7.3 Hz, 1H), 7.85 (t, *J* = 7.8 Hz, 1H), 7.79 (s, 1H), 7.72 to 7.67 (m, 2H), 7.40 to 7.32 (m, 5H), 67.22 to 7.13 (m, 3H), 6.58 (dd, *J* = 2.2 Hz, 8.7 Hz, 1H). LC–MS (ESI) [M + H]^+^: 455.

### (Z)-2-Hydroxy-5-(5-((3-oxobenzo[b]thiophen-2(3H)-ylidene)methyl)furan-2-yl)-*N*-phenylbenzamide (#02, SBI-4668)

This compound was prepared according to method C. *Orange solid* (0.065 g, 74%). ^1^H NMR (400 MHz, DMSO-d_6_): *δ* 10.50 (s, 1H), 8.36 (d, *J* = 2.3 Hz, 1H), 7.87 (dd, *J* = 2.3 Hz, 8.7 Hz, 1H), 7.82 to 7.75 (m, 3H), 7.75 (s, 1H), 7.71 to 7.65 (m, 3H), 7.37 to 7.32 (m, 4H), 7.14 (d, *J* = 3.7 Hz, 1H), 7.08 (t, *J* = 7.3 Hz, 1H), 7.06 (d, *J* = 8.7 Hz, 1H). ^13^C NMR (101 MHz, DMSO-d_6_): *δ* 186.9, 165.4, 158.4, 157.1, 149.4, 145.3, 138.3, 135.5, 130.3, 128.9, 128.8, 126.2, 126.1, 126.0, 125.8, 124.5, 124.2, 122.9, 120.7, 120.3, 119.9, 118.8, 118.1, 108.9. LC–MS (ESI) [M + H]^+^: 440. High-resolution MS (ESI) calculated for C_26_H_17_NO_4_S [M + H]^+^: 440.0872. Found: 440.0945.

### (Z)-2-Hydroxy-5-(5-((3-oxobenzo[b]thiophen-2(3H)-ylidene)methyl)furan-2-yl)-*N*′-phenylbenzohydrazide (#11, SBI-2128)

This compound was prepared according to method C. *Red solid* (0.052 g, 57%). ^1^H NMR (400 MHz, DMSO-d_6_): *δ* 12.16 (s, 1H), 10.55 (s, 1H), 8.44 (d, *J* = 2.3 Hz, 1H), 7.87 (d, *J* = 7.8 Hz, 2H), 7.81 (s, 1H), 7.74 (t, *J* = 7.3 Hz, 2H), 7.43 (d, *J* = 7.8 Hz, 1H), 7.37 (d, *J* = 3.7 Hz, 1H), 7.24 to 7.16 (m, 5H), 6.87 (d, *J* = 8.2 Hz, 1H), 6.76 (t, *J* = 6.9 Hz, 1H). LC–MS (ESI) [M + H]^+^: 455.

### (Z)-2-((5-(4-Hydroxy-3-(piperidine-1-carbonyl)phenyl)furan-2-yl)methylene)benzo[b]thiophen-3(2H)-one (#12, SBI-2131)

This compound was prepared according to method C. *Reddish orange solid* (0.037 g, 43%). ^1^H NMR (400 MHz, DMSO-d_6_): *δ* 8.36 (s, 1H), 7.78 (t, *J* = 7.8 Hz, 2H), 7.72 (d, *J* = 8.2 Hz, 1H), 7.71 (s, 1H), 7.67 (t, *J* = 7.3 Hz, 1H), 7.59 (s, 1H), 7.33 (t, *J* = 7.3 Hz, 1H), 7.27 (d, *J* = 3.7 Hz, 1H), 7.14 (d, *J* = 3.7 Hz, 1H), 7.01 (d, *J* = 8.2 Hz, 1H), 3.62 (brs, 4H), 1.52 (brs, 2H), 1.48 (brs, 4H). LC–MS (ESI) [M + H]^+^: 432.

### (Z)-2-Hydroxy-*N*-isopropyl-5-(5-((3-oxobenzo[b]thiophen-2(3H)-ylidene)methyl)furan-2-yl)benzamide (#13, SBI-0287)

This compound was prepared according to method C. *Red solid* (0.047 g, 58%). ^1^H NMR (400 MHz, DMSO-d_6_): *δ* 8.70 (d, *J* = 4.1 Hz, 1H), 8.37 (s, 1H), 7.92 (d, *J* = 8.2 Hz, 1H), 7.85 to 7.71 (m, 4H), 7.41 to 7.39 (m, 2H), 7.17 to 7.10 (m, 2H), 4.17 (m, 1H), 1.24 (d, *J* = 6.4 Hz, 6H). ^13^C NMR (101 MHz, DMSO-d_6_): *δ* 186.9, 167.3, 160.6, 157.0, 149.3, 145.3, 133.5, 130.2, 129.1, 126.3, 126.1, 125.8, 124.8, 124.4, 122.7, 119.9, 118.7, 118.3, 116.3, 108.8, 41.2, 22.1. LC–MS (ESI) [M + H]^+^: 406.

### (Z)-3-(5-((3-Oxobenzo[b]thiophen-2(3H)-ylidene)methyl)furan-2-yl)-*N*-phenylbenzamide (#15, SBI-5923)

This compound was prepared according to method C. *Orange solid* (0.056 g, 66%). ^1^H NMR (400 MHz, DMSO-d_6_): *δ* 10.41 (s, 1H), 8.38 (s, 1H), 8.07 (d, *J* = 7.8 Hz, 1H), 7.92 (d, *J* = 7.8 Hz, 1H), 7.81 (d, *J* = 7.8 Hz, 1H), 7.78 to 7.75 (m, 4H), 7.69 (t, *J* = 7.8 Hz, 2H), 7.42 (d, *J* = 3.7 Hz, 1H), 7.36 to 7.32 (m, 4H), 7.09 (t, *J* = 8.7 Hz, 1H). ^13^C NMR (101 MHz, DMSO-d_6_): *δ* 187.0, 165.1, 156.2, 150.2, 145.3, 139.0, 136.1, 135.7, 130.1, 129.5, 129.1, 128.6, 127.9, 127.2, 126.8, 126.3, 125.9, 124.5, 123.8, 123.7, 122.3, 120.4, 118.6, 110.9. LC–MS (ESI) [M + H]^+^: 424.

### (Z)-*N*-benzyl-3-(5-((3-oxobenzo[b]thiophen-2(3H)-ylidene)methyl)furan-2-yl)benzamide (#16, SBI-2129)

This compound was prepared according to method C. *Reddish orange solid* (0.055 g, 63%). ^1^H NMR (400 MHz, DMSO-d_6_): *δ* 9.59 (brs, 1H), 8.42 (d, *J* = 1.8 Hz, 1H), 7.92 (dd, *J* = 1.8 Hz, 8.2 Hz, 1H), 7.83 (d, *J* = 7.8 Hz, 1H), 7.77 (s, 1H), 7.76 (d, *J* = 7.8 Hz, 1H), 7.71 (t, *J* = 8.4 Hz, 1H), 7.39 to 7.32 (m, 8H), 7.12 (d, *J* = 3.7 Hz, 1H), 7.08 (d, *J* = 8.7 Hz, 1H), 4.57 (d, *J* = 5.5 Hz, 2H). ^13^C NMR (101 MHz, DMSO-d_6_): *δ* 186.8, 167.9, 156.3, 149.3, 145.3, 138.9, 135.4, 130.3, 129.2, 128.4, 127.3, 126.9, 126.2, 125.8, 125.1, 124.4, 122.8, 118.7, 118.6, 116.7, 108.6, 42.4. LC–MS (ESI) [M + H]^+^: 438.

### (Z)-2-Methoxy-5-(5-((3-oxobenzo[b]thiophen-2(3H)-ylidene)methyl)furan-2-yl)-*N*-phenylbenzamide (#18, SBI-2349)

This compound was prepared according to method C. *Reddish orange solid* (0.053 g, 58%). ^1^H NMR (400 MHz, DMSO-d_6_): *δ* 10.29 (s, 1H), 8.12 (d, *J* = 1.8 Hz, 1H), 8.05 (dd, *J* = 1.8 Hz, 8.7 Hz, 1H), 7.86 (d, *J* = 7.8 Hz, 1H), 7.80 (s, 1H), 7.78 to 7.70 (m, 4H), 7.42 to 7.34 (m, 6H), 7.13 (t, *J* = 6.9 Hz, 1H), 3.97 (s, 3H). ^13^C NMR (101 MHz, DMSO-d_6_): *δ* 186.9, 164.0, 156.9, 156.6, 145.5, 145.2, 139.0, 135.5, 130.3, 128.7, 127.6, 126.5, 126.3, 125.9, 125.7, 124.5, 123.6, 122.9, 121.5, 119.6, 118.8, 112.9, 109.4, 56.2. LC–MS (ESI) [M + H]^+^: 454.

### (Z)-2-((5-(4-Hydroxy-3-(piperazine-1-carbonyl)phenyl)furan-2-yl)methylene)benzo[b]thiophen-3(2H)-one (#19, SBI-2124)

This compound was prepared according to method C. *Reddish orange solid* (0.046 g, 53%). ^1^H NMR (400 MHz, DMSO-d_6_): *δ* 8.18 (s, 1H), 8.01 (s, 1H), 7.80 to 7.74 (m, 3H), 7.72 (s, 1H), 7.68 (d, *J* = 7.3 Hz, 1H), 7.76 (d, *J* = 1.8 Hz, 1H), 7.36 (t, *J* = 7.3 Hz, 1H), 7.29 (d, *J* = 3.7 Hz, 1H), 7.17 (d, *J* = 3.7 Hz, 1H), 7.03 (d, *J* = 8.2 Hz, 1H), 3.36 to 3.32 (m, 4H), 3.28 to 3.25 (m, 4H). ^13^C NMR (101 MHz, DMSO-d_6_): *δ* 187.3, 166.7, 157.9, 155.1, 149.6, 145.1, 135.9, 130.8, 127.0, 126.7, 125.4, 125.3, 125.0, 123.5, 120.7, 119.3, 117.1, 109.2, 45.9, 44.8. LC–MS (ESI) [M + H]^+^: 433.

### (Z)-*N*-isopropyl-3-(5-((3-oxobenzo[b]thiophen-2(3H)-ylidene)methyl)furan-2-yl)benzamide (#20, SBI-9639)

This compound was prepared according to method C. Orange solid (0.049 g, 63%). ^1^H NMR (400 MHz, DMSO-d_6_): *δ* 8.33 (d, *J* = 7.3 Hz, 1H), 8.27 (s, 1H), 7.99 (d, *J* = 7.8 Hz, 1H), 7.81 (d, *J* = 7.8 Hz, 2H), 7.78 (s, 1H), 7.76 (d, *J* = 7.8 Hz, 1H), 7.69 (t, *J* = 7.3 Hz, 1H), 7.69 (t, *J* = 7.3 Hz, 1H), 7.38 to 7.33 (m, 3H), 4.12 to 4.07 (m, 1H), 1.14 (d, *J* = 6.4 Hz, 6H). ^13^C NMR (101 MHz, DMSO-d_6_): *δ* 187.0, 164.8, 156.4, 150.1, 145.3, 139.9, 135.7, 130.1, 129.2, 128.8, 127.7, 127.1, 126.3, 125.9, 124.5, 123.4, 122.4, 118.7, 110.7, 41.1, 22.3. LC–MS (ESI) [M + H]^+^: 390.

### (Z)-2-Hydroxy-*N*-(2-hydroxyethyl)-5-(5-((3-oxobenzo[b]thiophen-2(3H)-ylidene)methyl)furan-2-yl)benzamide (#22, SBI-1457)

This compound was prepared according to method C. *Reddish orange solid* (0.036 g, 44%). ^1^H NMR (400 MHz, DMSO-d_6_): *δ* 8.99 (t, *J* = 5.6 Hz, 1H), 8.43 (d, *J* = 2.3 Hz, 1H), 7.94 (dd, *J* = 8.6, 2.2 Hz, 1H), 7.85 (dd, *J* = 7.9, 1.3 Hz, 1H), 7.84 to 7.81 (m, 1H), 7.80 (s, 1H), 7.75 to 7.70 (m, 1H), 7.40 (td, *J* = 7.4, 1.0 Hz, 1H), 7.37 (d, *J* = 3.8 Hz, 1H), 7.17 (d, *J* = 3.7 Hz, 1H), 7.12 (d, *J* = 8.6 Hz, 1H), 4.88 (s, 1H), 3.60 to 3.57 (m, 2H), 3.45 to 3.41 (m, 2H). LC–MS (ESI) [M + H]^+^: 408.

### (Z)-2-((5-(4-Hydroxy-3-(4-methylpiperazine-1-carbonyl)phenyl)furan-2-yl)methylene)benzo[b]thiophen-3(2H)-one (#23, SBI-2125)

This compound was prepared according to method C. *Reddish orange solid* (0.052 g, 58%). ^1^H NMR (400 MHz, DMSO-d_6_): *δ* 7.86 to 7.68 (m, 6H), 7.40 (t, *J* = 7.3 Hz, 1H), 7.34 (d, *J* = 3.7 Hz, 1H), 7.23 (d, *J* = 3.7 Hz, 1H), 7.02 (d, *J* = 8.2 Hz, 1H), 3.43 (brs, 4H), 2.31 (brs, 4H), 2.19 (s, 3H). LC–MS (ESI) [M + H]^+^: 447.

### (Z)-2-Fluoro-5-(5-((3-oxobenzo[b]thiophen-2(3H)-ylidene)methyl)furan-2-yl)-*N*-phenylbenzamide (#24, SBI-2348)

This compound was prepared according to method C. *Reddish orange solid* (0.052 g, 59%). ^1^H NMR (400 MHz, DMSO-d_6_): *δ* 10.61 (s, 1H), 8.43 (s, 1H), 8.20 (dd, *J* = 2.8 Hz, 7.6 Hz, 1H), 7.86 (d, *J* = 7.8 Hz, 1H), 7.80 (s, 1H), 7.78 to 7.70 (m, 5H), 7.59 (t, *J* = 9.2 Hz, 1H), 7.45 (d, *J* = 3.7 Hz, 1H), 7.43 to 7.34 (m, 3H), 7.15 (t, *J* = 7.3 Hz, 1H). LC–MS (ESI) [M + H]^+^: 442.

### Reagents and compounds

RMC-4550 was purchased from ProbeChem. The IRS-1 peptide was synthesized by PepMic. Reagents and buffer components were purchased from Thermo Fisher Scientific unless noted.

### Protein expression and purification

Recombinant human full-length SHP2 (1–594) WT and E76K mutants, as well as the SHP2cat (248–527), were expressed and purified as described before (36, 41). Recombinant human PTP1B (1–300) and the codon-optimized STEP (280–566) catalytic domains were cloned into PET-15b and expressed as *N*-His-tagged fusion proteins. For expression, transformed BL21(DE3) cells were grown and induced similar as described for SHP2. Collected cells were resuspended in lysis buffer (25 mM Tris, pH 7.5, 300 mM NaCl, 50 mM imidazole, and 10% glycerol) with 100 mg/l RNaseA and were lysed with two passages using a microfluidizer. The lysate was clarified by centrifugation at 15,000*g* for 50 min and applied to nickel–nitrilotriacetic acid resin. The column resin was washed, and the PTP1B or STEP protein was eluted in lysis buffer at 300 mM imidazole. The PTP1B or STEP protein was further purified by S75 size-exclusion chromatography in 50 mM Tris, pH 7.5, 50 mM NaCl. The eluted peak fractions were supplemented with tris(2-carboxyethyl)phosphine to 10 mM, concentrated by ultrafiltration, and stored at −80 °C. The purified yield of PTP1B was 33 mg/l cell culture; the yield of STEP was 16 mg/l cell culture.

### SHP2, PTP1B, and STEP biochemical inhibition assays

SHP2 inhibitors were tested at RT in a 384-well plate format standard phosphatase fluorescence intensity assay using DiFMUP (Invitrogen/Thermo Fisher Scientific) as a substrate and a total reaction volume of 25 μl. SHP2 inhibitors or vehicle (DMSO) were spotted in triplicate into a black Greiner FLUOTRAC 200 384-well microplate (catalog no.: 781076; Greiner) for a 10-point dose–response assay using an Echo 555 Liquid Handler (Labcyte, Inc). PTP working solutions were prepared at a 0.625 nM concentration (for a final concentration of 0.5 nM) in buffer containing 50 mM Bis–Tris, pH 6.0, 50 mM NaCl, 5 mM DTT, and 0.01% Tween-20. Prior to the assay, a dually phosphorylated IRS-1 peptide (625 nM [500 nM final] (30)) was added to full-length WT SHP2 working solutions and incubated for 20 min. DiFMUP working solutions at 5× final concentration were prepared in 50 mM Bis–Tris, pH 6.0, 50 mM NaCl, and 0.01% Tween-20. About 20 μl of PTP working solution was dispensed into the microplate and incubated with inhibitor for 20 min at RT. About 5× DiFMUP working solutions were prepared for final concentrations corresponding to the respective *K*_*m*_ value for each protein (SHP2-WT, 60 μM; SHP2-E76K, 20 μM; SHP2cat, 20 μM; PTP1B, 25 μM; and STEP, 4 μM). The reaction was initiated by addition of 5 μl DiFMUP working solutions. Fluorescence intensity was measured in kinetic mode (every minute for 7 or 10 min) using a Tecan Spark Multimode Microplate Reader (Tecan) with an excitation wavelength of 360 nm and an emission wavelength of 460 nm. The initial rates were determined from the linear progression curves of the PTP reaction. The nonenzymatic hydrolysis of the substrate was corrected by using a control without addition of enzyme. IC_50_ values were calculated from the corrected initial rates by nonlinear regression using the program GraphPad Prism, version 8 (GraphPad Software, Inc) software. Dose–response inhibition assays of SHP2cat using the substrate OMFP were performed similarly as described previously for DiFMUP. OMFP was used at a concentration corresponding to its *K*_*m*_ value for SHP2cat (50 μM final). Fluorescence intensity was measured in kinetic mode (every minute for 10 min) using a Tecan Spark Multimode Microplate Reader with an excitation wavelength of 485 nm and an emission wavelength of 535 nm. Initial rates were determined, and IC_50_ values were calculated as described for the DiFMUP assay above.

### Michaelis–Menten kinetics and mode of inhibition determination

SBI-4668 was tested with SHP2-E76K using a similar assay format as described previously. SBI-4668 or vehicle (DMSO) was spotted in triplicate into a black Greiner FLUOTRAC 200 384-well microplate (catalog no.: #781076; Greiner) using an Echo 555 Liquid Handler (Labcyte, Inc). SHP2-E76K working solution was prepared and dispensed as described previously and incubated with inhibitor for 20 min at RT. DiFMUP working solutions at 5× final concentration were prepared in 50 mM Bis–Tris, pH 6.0, 50 mM NaCl, and 0.01% Tween-20. The reaction was initiated by addition of 5 μl DiFMUP working solutions for final DiFMUP concentrations of 100, 50, 25, 12.5, 6.25, and 3.125 μM. Fluorescence intensity was measured in kinetic mode as described previously. The initial rates were determined from the linear progression curves of each SHP2-E76K reaction. The nonenzymatic hydrolysis of the substrate was corrected for each DiFMUP concentration by using a control without addition of enzyme. Michaelis–Menten plots were generated for each inhibitor concentration using nonlinear regression and fitting initial rates to the Michaelis–Menten equations for competitive, noncompetitive, uncompetitive, or mixed inhibition using the GraphPad Prism, version 8, software. For a comparison of the fitting results, the second-order corrected Akaike's Information Criterion (AICc) was calculated using Equation 1, where N is the number of data points, SS the absolute sum of squares, and K the number of parameters fit by nonlinear regression plus 1.

(1) $${\text{AIC}}_{\text{c}}\;=\text{ Nln}\left(\text{SS}/\text{N}\right)+\text{2K}+\left(2\text{K}\left(\text{K}+1\right)\right)/\left(\text{N}-\text{K}-1\right)$$

The probability for one mode of inhibition compared with another one was computed by using Equation 2, where Δ is the difference between AICc scores of the two models being compared.

(2) Probability = exp(−0.5Δ)/(1+exp(−0.5Δ))

### Jump-dilution inhibition assay

SBI-2130 was tested for reversibility of inhibition in a jump-dilution experiment using SHP2cat. A regular dose–response experiment (no jump dilution) was performed in parallel. Working solutions of SBI-2130 in DMSO included 10, 3, 1, 0.3, 0.1, 0.03, 0.01, and 0 mM for the jump-dilution experiment and 1, 0.3, 0.1, 0.03, 0.01, 0.003, 0.001, and 0 mM for the regular dose–response experiment. SHP2cat working solutions were prepared at concentrations of 6.25 nM for the jump-dilution experiment, and 0.625 nM for the regular dose–response experiment, in buffer containing 50 mM Bis–Tris, pH 6.0, 50 mM NaCl, 0.5 mM EDTA, 5 mM DTT, and 0.01% Tween-20. Separate tubes were prepared for each experimental condition, containing 99 μl of the respective SHP2cat working solution and 1 μl compound solution. SHP2cat and compound were incubated for 10 min at RT, before 900 μl buffer was added to the jump-dilution experiment tubes, resulting in a 10× dilution. SHP2cat/compound mixtures were incubated for another 10 min at RT, before 20 μl of each solution was transferred into a black Greiner FLUOTRAC 200 384-well microplate (catalog no.: 781076; Greiner) for a quadruplicate measurement. The enzyme reaction was started by the addition of 5 μl of DiFMUP working solution (100 μM in 50 mM Bis–Tris, pH 6.0, 50 mM NaCl, 0.5 mM EDTA, and 0.01% Tween-20), and fluorescence intensity was measured in kinetic mode (every minute for 10 min) using a Tecan Spark Multimode Microplate Reader as described previously. The final concentrations were as follows: SHP2cat, 0.5 nM; DiFMUP, 20 μM; SBI-2130, 8, 2.4, 0.8, 0.24, 0.08, 0.024, 0.008, and 0 μM. The initial rates were determined from the linear progression curves of the SHP2cat reaction. The nonenzymatic hydrolysis of the substrate was corrected by using a control without addition of enzyme. IC_50_ values were calculated from the corrected initial rates by nonlinear regression using the program GraphPad Prism, version 9.

### PTS assay

Differential scanning fluorimetry (also known as protein thermal shift) measurements of SHP2cat were performed using optimized methods and conditions in accordance with those previously described (36, 41, 49). SBI-4668 was spotted at different concentrations into MicroAmp 384-well real-time PCR plates (catalog no.: 4483285; Applied Biosystems) using an Echo 555 Liquid Handler. About 5 μl of SHP2cat working solution (1.5 μM in 50 mM Tris–HCl, pH 7.5, 50 mM NaCl, and 5 mM DTT) was added to each well using a Multidrop Combi Reagent Dispenser (Thermo Fisher Scientific). About 5 μl of 5× SYPRO Orange (Invitrogen/Thermo Fisher Scientific) dissolved in molecular-grade water was equally dispensed into the PCR plate wells, diluting the enzyme solution 1:2. The plate was then sealed with MicroAmp Optical Adhesive Film (Applied Biosystems) and spun to collect the reaction mix at the bottom of the plate. Plates were analyzed using a ViiA 7 Real-Time PCR instrument (Applied Biosystems) and a 12 min temperature gradient with a temperature increase of 0.075 °C/s. The melting temperature and thermal profiles were determined as described previously using Protein Thermal Shift Software, version 1.3 (Applied Biosystems) (36, 49).

### Cell culture

The AML cell lines MOLM-13 and Kasumi-1 and the esophageal carcinoma cell line KYSE-520 were obtained from DSMZ (German Collection of Microorganisms and Cell Cultures). AML cell lines MV4-11 and U937 were obtained from American Type Culture Collection. Cell lines were cultured in RPMI 1640 media with l-glutamine (Corning) supplemented with 10% fetal bovine serum (FBS) (Gibco) and 1% antibiotic–antimycotic solution (Gibco). Cells were incubated in flasks at 37 °C and 5% CO_2_.

Patient-derived AML cells were obtained from Carol Burian and Dr James Mason (Scripps MD Anderson Center) under approved Institutional Review Board protocol 13-6180. Peripheral blood mononuclear cells were isolated by traditional Ficoll–Paque PLUS (17-1440-02; GE Healthcare) centrifugation according to the manufacturer's instructions, and red blood cells were lysed using RBC lysis buffer (catalog no.: J62990; Alfa Aesar). Final peripheral blood mononuclear cells were resuspended in Bambanker serum-free freezing medium (Wako Pure Chemical Industries, Ltd) and stored frozen before cell viability was assessed as described later.

### Cell viability assays

Viability of AML and esophageal carcinoma cells was assessed using the ATP-depletion assay CellTiter-Glo (Promega). Cells were harvested at 1 × 10^6^/ml to 2 × 10^6^ cells/ml with cell numbers determined by trypan blue using the Countess Cell counter (Thermo Scientific) and resuspended in culture media. About 3000 cells (in 20 μl) were seeded in a 384-well format white and clear-bottom microplate (catalog no.: 781098; Greiner) spotted with test compound or vehicle control (DMSO) using an Echo 555 Liquid Handler. Cells were incubated for 3 days, before 10 μl of CellTiter-Glo reagent mix was added to each well and incubated for 10 min at RT. Luminescence was read on a Tecan Spark Multimode Microplate Reader, and data were analyzed using GraphPad Prism software.

Triple-negative breast cancer cell viability was assessed using the ATP-depletion assay CellTiter-Glo. Briefly, 2500 cells in 25 μl media (RPMI + 10% FBS + 1× penicillin–streptomycin [Omega Scientific]) were seeded per well of a 384-well tissue culture–treated plate (Greiner). About 25 nl of 1000× test compound were added using a Labcyte Echo acoustic dispenser, and the cells were incubated for 5 days before addition of 10 μl of CellTiter-Glo reagent as described by the manufacturer. Luminescence was detected on a BioTek Synergy 2 microplate reader, and the values normalized to those of vehicle (DMSO)-treated controls before being plotted using GraphPad Prism.

### Cell colony formation assays

MDA-MB-468 and BT-459 triple-negative breast cancer cells were maintained in Dulbecco's modified Eagle's medium + 10% FBS supplemented with 1× penicillin–streptomycin/l-glutamine (Omega Scientific). About 1500 cells (in 300 μl) were seeded per well of a standard 24-well tissue culture plate (Falcon) and allowed to adhere for 24 h, before the addition of compound(s) as described. After a further 96 h, media were replaced with fresh media containing the same test compounds and concentrations. After 11 days, media were removed, and cells stained with 0.5% crystal violet (in 20% methanol; Sigma) for 20 min with agitation at 80 rpm. Stain was removed, and plates with stained cells were washed by being submerged in excess deionized water and allowed to air dry overnight before imaging.

### Immunoblot assays

Cells cultured in RPMI medium were treated as indicated in the legends to the figures. Total protein extracts were prepared in modified radioimmunoprecipitation assay lysis buffer (25 mM Tris–HCl, pH 7.4, 10% glycerol, 0.2% Triton X-100, 150 mM NaCl, 2 mM Na_3_VO_4_, and 1 mM EDTA) containing a protease inhibitor cocktail (Roche Applied Science). Equal amounts of protein were separated on 4 to 12% (p-ERK1/2 blots) or 4 to 20% (SHP2 blots) Bis–Tris gels by SDS-PAGE and transferred to nitrocellulose (p-ERK1/2 blots) or 0.2 μm polyvinylidene fluoride (SHP2 blots) membranes. Membranes were blocked in 5% dry milk (Bio-Rad) in Tris-buffered saline–Tween-20 (TBS–T) (0.1% v/v) for 1 h at RT. Protein-bound membranes were incubated with indicated primary antibody overnight at 4 °C. After washing three times with TBS-T, membranes were incubated for 1 h with horseradish peroxidase–conjugated secondary antibody and then visualized by an ECL Prime detection system (GE Healthcare). Immunoblotting experiments were carried out at least three times, and representative images are shown. Densitometric quantitation of immunoblots was performed using Image Studio Lite Software (LI-COR). Changes of phosphorylated ERK1/2 to total ERK1/2 ratios are expressed as percentage of the vehicle-treated controls. The following antibodies were used: Phospho-Erk1/2 Ab (catalog no.: 9101S; Cell Signaling); total Erk1/2 Ab (catalog no.: 9102S; Cell Signaling); SHP2 Ab (catalog no.: A301-544A; lot no.: 1; Bethyl); and GAPDH (14C10) mAb (catalog no.: 2118S; lot no.: 14; Cell Signaling).

### SHP2 CRISPR–Cas9 KO and inhibitor selectivity assays

High-efficiency Cas9-editing MOLM-13 cells were generated by transducing MOLM-13 cells with the pLenti-Cas9-blasticidin construct (Addgene plasmid no. 52962 from Dr Feng Zhang) and selecting single stable clones using flow sorting. Clones were then tested for editing efficiency by performing tracking of indels by decomposition analysis (45). These MOLM13-Cas9 cells were then transduced with a lentiviral construct containing an AAVS sgRNA and an mCherry reporter out of frame and downstream of an AAVS sgRNA targeting site. The cells were bulk sorted for mCherry+ expression by flow cytometry using a BD FACSAria Cell Sorter II (BD Biosciences), indicative of successful AAVS editing, and used in subsequent experiments.

For SHP2 KO, dual sgRNAs targeting SHP2 were cloned in the pLentiGuide puro vector (Addgene plasmid no. 52963 from Dr Feng Zhang) using a published protocol (50). This SHP2 dual sgRNA plasmid was used to make virus using standard protocols with pPAX2 and pMD.2 as packaging vectors. The virus was used to stably transduce MOLM13-Cas9-mCherry cells, and cells were selected using puromycin (Gibco; 1 μg/ml). SHP2 KO was evaluated by immunoblotting. SHP2 inhibitors were tested in parallel in regular MOLM-13 cells expressing SHP2 and in MOLM-13-Cas9-mCherry cells with SHP2 KO. Experiments with regular MOLM-13 cells were performed as described previously. For SHP2 KO, MOLM-13-Cas9-mCherry cells were seeded in nontreated 6-well plates (2 × 10^6^ cells/well in 2.5 ml), with cell numbers determined by trypan blue using the Countess Cell Counter (Thermo Scientific). Cells were transduced with 10 μl of SHP2 dual sgRNA lentivirus added to the culture media. Polybrene (Sigma–Aldrich; 10 mg/ml; TR-1003-G) at a concentration of 0.8 μg/ml was added to experimental wells. Cells were incubated overnight at standard cell culture conditions, before spun down and resuspended in fresh media (RPMI 1640 with l-glutamine + 10% FBS + 1% antibiotic–antimycotic solution + 1% l-glutamine solution). Puromycin (10 mg/ml) at a concentration of 2.5 μg/ml was added to the cells 48 h after transduction for 3 days. About 3000 cells/well (in 20 μl) were seeded in a 384-well white and clear-bottom microplate (catalog no.: 781098; Greiner) and spotted with test compounds or vehicle control (DMSO) using an Echo 555 Liquid Handler. Cells were incubated for 3 days at standard cell culture conditions. Cell viability was assessed using the ATP-depletion assay CellTiter-Glo as described previously, and data were analyzed using GraphPad Prism software.

## Data availability

All data are contained within the manuscript.

## Conflict of interest

The authors declare that they have no conflicts of interest with the contents of this article.
